# Progressive exercise compared with best practice advice, with or without corticosteroid injection, for the treatment of patients with rotator cuff disorders (GRASP): a multicentre, pragmatic, 2 × 2 factorial, randomised controlled trial

**DOI:** 10.1016/S0140-6736(21)00846-1

**Published:** 2021-07-31

**Authors:** Sally Hopewell, David J Keene, Ioana R Marian, Melina Dritsaki, Peter Heine, Lucy Cureton, Susan J Dutton, Helen Dakin, Andrew Carr, Willie Hamilton, Zara Hansen, Anju Jaggi, Chris Littlewood, Karen L Barker, Alastair Gray, Sarah E Lamb, GRASP Trial Group, Marcus Bateman, Alison Hallett, Helen Thompson, Elaine Willmore, Lucy McCann, Jonathan Price, Neil Smith, Harry Kardamilas, Matt Hurst, Tim Andrews, Lori Wells, Chloe De Matas, Arun Jaykumar, Sean Grove, Corinne Birch, Julie Bury, James Blacknall, Sally Jessop, Llewelyn Boucher, Robert Sandbach, Stacey Lalande, Gill Dickson, Treena Larkin, Carole Cummings

**Affiliations:** a Nuffield Department of Orthopaedics, Rheumatology and Musculoskeletal Sciences, University of Oxford, Oxford, UK; b Nuffield Department of Population Health, University of Oxford, Oxford, UK; c College of Medicine and Health, University of Exeter, Exeter, UK; d Royal National Orthopaedic Hospital NHS Trust, Stanmore, Middlesex, UK; e Department of Health Professions, Manchester Metropolitan University, Manchester, UK

## Abstract

**Background:**

Corticosteroid injections and physiotherapy exercise programmes are commonly used to treat rotator cuff disorders but the treatments' effectiveness is uncertain. We aimed to compare the clinical effectiveness and cost-effectiveness of a progressive exercise programme with a single session of best practice physiotherapy advice, with or without corticosteroid injection, in adults with a rotator cuff disorder.

**Methods:**

In this pragmatic, multicentre, superiority, randomised controlled trial (2 × 2 factorial), we recruited patients from 20 UK National Health Service trusts. We included patients aged 18 years or older with a rotator cuff disorder (new episode within the past 6 months). Patients were excluded if they had a history of significant shoulder trauma (eg, dislocation, fracture, or full-thickness tear requiring surgery), neurological disease affecting the shoulder, other shoulder conditions (eg, inflammatory arthritis, frozen shoulder, or glenohumeral joint instability), received corticosteroid injection or physiotherapy for shoulder pain in the past 6 months, or were being considered for surgery. Patients were randomly assigned (centralised computer-generated system, 1:1:1:1) to progressive exercise (≤6 sessions), best practice advice (one session), corticosteroid injection then progressive exercise, or corticosteroid injection then best practice advice. The primary outcome was the Shoulder Pain and Disability Index (SPADI) score over 12 months, analysed on an intention-to-treat basis (statistical significance set at 1%). The trial was registered with the International Standard Randomised Controlled Trial Register, ISRCTN16539266, and EuDRACT, 2016-002991-28.

**Findings:**

Between March 10, 2017, and May 2, 2019, we screened 2287 patients. 708 patients were randomly assigned to progressive exercise (n=174), best practice advice (n=174), corticosteroid injection then progressive exercise (n=182), or corticosteroid injection then best practice advice (n=178). Over 12 months, SPADI data were available for 166 (95%) patients in the progressive exercise group, 164 (94%) in the best practice advice group, 177 (97%) in the corticosteroid injection then progressive exercise group, and 175 (98%) in the corticosteroid injection then best practice advice group. We found no evidence of a difference in SPADI score between progressive exercise and best practice advice when analysed over 12 months (adjusted mean difference −0·66 [99% CI −4·52 to 3·20]). We also found no evidence of a difference between corticosteroid injection compared with no injection when analysed over 12 months (−1·11 [–4·47 to 2·26]). No serious adverse events were reported.

**Interpretation:**

Progressive exercise was not superior to a best practice advice session with a physiotherapist in improving shoulder pain and function. Subacromial corticosteroid injection provided no long-term benefit in patients with rotator cuff disorders.

**Funding:**

UK National Institute for Health Research Technology Assessment Programme.

## Introduction

Shoulder pain is common, with approximately 1% of adults aged 45 years and older presenting to primary care services with a new episode of shoulder pain each year,^1^ accounting for 2·4% of all general practitioner (GP) consultations in the UK.^2^ Disorders of the rotator cuff are the most common cause, accounting for 70% of cases.^1^ Rotator cuff disorders are often associated with substantial and persistent disability and pain and approximately half of patients continue to have pain or functional limitations for up to 2 years.^2^ Most problems with shoulder pain are managed in primary care by physiotherapists and GPs. The aim of treatment is to improve pain and shoulder function. Treatment options include rest, advice, analgesia, non-steroidal anti-inflammatory drugs, exercise, manual therapy, and corticosteroid injections.^3^

**Research in context**

**Evidence before this study**

A Cochrane review published in 2016 highlighted insufficient evidence about the long-term clinical and cost-effectiveness of physiotherapy for the treatment of patients with rotator cuff disorders despite the treatment's widespread provision. Evidence from several small trials with short-term follow-up also raised uncertainty about which types of exercise and delivery mechanisms were associated with best outcomes. We searched MEDLINE, Embase, and CINAHL to identify new evidence relevant to the GRASP (Getting it Right: Addressing Shoulder Pain) trial (date of last search June 30, 2020; see appendix p 20 for search terms). From an initial 2354 records, we identified seven trials published between 2013 and 2020, comparing the effects of supervised exercise versus unsupervised exercise, or no intervention, in people with a rotator cuff disorder (excluding those who required surgery). The quality of trials was variable and considerable heterogeneity meant we could not combine data from studies. All trials were small apart from two moderate sized trials. Only two of the seven trials reported on the effect of exercise on shoulder pain and function at 12 months. Most of the trials concluded that little or no difference existed between supervised and unsupervised exercise at 6 and 12 months, with the exception of the SUPPORT trial, which showed a benefit of supervised exercise compared with an exercise leaflet at 6 months but not at 12 months. At the time of planning the GRASP trial, a systematic review showed that in comparison with placebo, corticosteroid injection had short-term benefit for treating patients with tendinopathy, although uncertainty existed regarding the injection's use in patients with rotator cuff disorders. We searched MEDLINE, Embase, Allied and Complementary Medicine Database, CINAHL, the Cochrane Central Register of Controlled Trials, ClinicalTrials.gov, and the WHO International Clinical Trials Registry to identify new evidence relevant to the GRASP trial (date of last search June 30, 2020; see appendix p 20 for search terms). From an initial 794 records, we identified one small trial (n=50) comparing the effects of corticosteroid injection versus no injection in patients with a rotator cuff tear, which found no difference in shoulder pain and function when analysed at 3 months or 6 months. We identified an additional ten trials comparing the effects of corticosteroid injection with placebo injection, of which four were judged as suitable for inclusion in a meta-analysis. The remaining trials could not be included because of either incomplete or incompatible outcome data. In these trials, corticosteroid injection showed a benefit over placebo in the short term but not the medium term for shoulder pain and function. No trials provided outcome data beyond 6-month follow-up and none reported any serious adverse events as a result of injection.

**Added value of this study**

To our knowledge, the GRASP trial is the largest randomised controlled trial to date that has assessed the effects of exercise interventions, with or without the use of subacromial corticosteroid injection, in adults with a new episode of shoulder pain attributable to a rotator cuff disorder. Our findings during 12-month follow-up showed that the progressive exercise intervention was not superior to a best practice advice session with a physiotherapist. Subacromial corticosteroid injection was not superior to having no injection during 12-month follow-up, apart from modest improvement in shoulder pain and function at 8 weeks; the greatest benefit being in those with worse pain and functional impairment. The health economic comparison found that best practice advice in combination with corticosteroid injection is expected to be the most cost-effective, although some uncertainty remains around this conclusion.

**Implications of all the available evidence**

The GRASP trial shows that a single face-to-face session with a physiotherapist is likely to be more cost-effective and is not significantly different in terms of clinical outcomes when compared with a comprehensive physiotherapy intervention of up to six face-to-face sessions. This finding is particularly important given the incidence of rotator cuff disorders and the need to develop cost-effective and pragmatic methods of dealing with this high volume of conditions. Subacromial corticosteroid injection provides a modest short-term but no long-term benefit, as seen in other trials, and was associated with participants being more likely to report doing their exercises as advised.

Evidence from small, short-term trials suggests that physiotherapist-prescribed exercise is promising. However, a Cochrane review highlighted the insufficient evidence about the treatment's long-term clinical effectiveness and cost-effectiveness.^4^ Despite widespread provision, uncertainty exists about which types of exercise and levels of physiotherapy supervision are associated with the best outcomes. This evidence is limited by problems in study design and lack of comparator groups.^5^, ^6^ Progressive resistance training to improve muscular strength, whether supervised or home based, has been identified as a core component of exercise for patients with rotator cuff disorders.^7^ Subacromial corticosteroid injections are commonly used to reduce local tissue inflammation and pain. Compared with placebo, corticosteroid injections have short-term benefit in the shoulder,^8^ although the longer-term benefits and harms are not known.^9^ Corticosteroid injections are being used increasingly in clinical practice alongside physiotherapy for the management of people with rotator cuff disorders;^3^ hence justification for investigating corticosteroid injection in the GRASP (Getting it Right: Addressing Shoulder Pain) trial alongside physiotherapist-prescribed exercise.

The aim of the GRASP trial was to compare the clinical effectiveness and cost-effectiveness of two interventions in adults with a new episode of shoulder pain attributable to a rotator cuff disorder: (1) an individually tailored, progressive exercise programme prescribed and supervised by a physiotherapist versus a best practice advice session with a physiotherapist; and (2) subacromial corticosteroid injection versus no injection.

## Methods

### Study design and participants

We did a multicentre, pragmatic, superiority, randomised controlled trial using a 2 × 2 factorial design. Patients aged 18 years or older were recruited from 20 UK National Health Service (NHS) trusts. Patients were eligible if they had a diagnosis of shoulder pain attributable to a rotator cuff disorder (eg, cuff tendonitis, impingement syndrome, tendinopathy, or rotator cuff tear) that had started within the past 6 months. We used the diagnostic criteria set out in the British Elbow and Shoulder Society (BESS) guidelines.^3^ Patients were excluded if they had a history of significant shoulder trauma (eg, dislocation, fracture, or full-thickness tear requiring surgery), neurological disease affecting the shoulder, other shoulder conditions (eg, inflammatory arthritis, frozen shoulder, or glenohumeral joint instability), received corticosteroid injection or physiotherapy for shoulder pain in the past 6 months, or were being considered for surgery. Detailed criteria are in the protocol.^10^ After patients were assessed for eligibility, informed written consent was obtained from all trial participants by a research facilitator trained in Good Clinical Practice at each participating site. Ethics approval was obtained from the National Research Ethics Service (NRES Berkshire B Research Ethics Committee: 16/SC/0508). The trial protocol,^10^ statistical analysis plan (SAP),^11^ and health economics analysis plan have been published previously.^12^

### Randomisation and masking

Consented participants were randomly assigned to one of four groups: (1) progressive exercise programme (a progressive home exercise programme that was individually tailored and prescribed and supervised by a physiotherapist involving up to six face-to-face sessions over 16 weeks), (2) best practice advice (one face-to-face session with a physiotherapist and home exercise programme supported by high quality self-management materials); (3) progressive exercise programme preceded by a subacromial corticosteroid injection; or (4) best practice advice session preceded by a subacromial corticosteroid injection.

Randomisation (1:1:1:1) was done by an independent research facilitator using the centralised computer randomisation service provided by the Oxford Clinical Trials Research Unit once the patient was enrolled and baseline questionnaire completed. Randomisation was computer generated and stratified by site, age (18–35 years, >35 years) and sex, using variable block sizes. We could not mask physiotherapists or study participants once treatment allocation was revealed. When practical, team members were masked until after data analysis was complete. Trial statisticians had access to treatment assignment during the study for the purposes of data monitoring and safety. Data entry personnel entered data from anonymised questionnaires, which included some details on treatments received.

### Procedures

Subacromial corticosteroid injection was delivered before the progressive exercise or best practice advice intervention and given predominately by extended-scope physiotherapists with an appropriate post-registration qualification. Appointments were coordinated so that participants would receive their injection within 10 days of randomisation. In accordance with the trial protocol, the corticosteroid injected was either methylprednisolone acetate (≤40 mg) or triamcinolone acetonide (≤40 mg), as per local treatment protocols. The local anaesthetic was either 1·0% lidocaine (≤5 mL) or 0·5% bupivacaine hydrochloride (≤10 mL). The choice and dose of corticosteroid and local anaesthetic (including volume), including the injection site, was recorded for each participant. A second injection could be given after 6 weeks in accordance with the trial protocol for patients who received good initial benefit from their first injection. Because of existing evidence of the greater effectiveness of corticosteroid injection compared with placebo for short-term management, we excluded the use of placebo injection from our protocol. Full details of the exercise interventions have been reported previously^13^ and here described in brief.

Participants randomly assigned to the progressive exercise intervention received up to six individual face-to-face sessions with a physiotherapist over 16 weeks. These sessions included a behavioural component to encourage adherence to the exercises. We chose the number of sessions, spread over this time, to enable progression of the intensity of exercise and a sufficient time for a physiological response in the neuromuscular system. Appointments were coordinated so that participants would start their first session within 14–28 days of randomisation. The initial session allowed up to 60 min for an initial examination of the shoulder and initial prescription of exercises. Thereafter, patients received up to five sessions, each 20–30 min, for the physiotherapist to progress or regress the exercise prescription using a standardised protocol.^13^ Exercises focused on movements commonly affected by a rotator cuff disorder: resisted external rotation, flexion, and abduction of the shoulder. Participants were given a folder containing an advice booklet, exercise action planner, diary, and instructions on their exercise programme set up in collaboration with the physiotherapist. Resistance bands were issued as required. Participants were asked to do their exercises 5 days per week, with two non-consecutive recovery days. Physiotherapists recorded the number of treatment sessions attended by each participant. The intervention was designed to support participants through a progressive dose of exercises and to optimise adherence to the home exercise plan.

Participants randomly assigned to the best practice advice intervention received a single individual face-to-face session with a physiotherapist, lasting up to 60 min. As per the progressive exercise intervention, appointments were coordinated so that participants would attend their session within 14–28 days of randomisation. Participants also received the same initial shoulder examination and advice booklet (with exercise action planner, diary, and resistance band) but the exercise programme was different. Participants were given a simple set of self-guided exercises (with access to exercise videos) that could be progressed and regressed depending on their capability. The exercises were designed using similar concepts to the progressive exercise intervention, such as increased resistance and done five times per week, but these were a simpler range of exercise options that were not supervised.^13^

Physiotherapists delivering progressive exercise and best practice advice were trained separately by a GRASP trial research physiotherapist and had access to a comprehensive intervention manual. A rigorous quality control programme was done to ensure intervention fidelity.

All participants were advised they could take over-the-counter analgesia as required, in accordance with the BESS guidelines.^3^ Participants could seek other forms of treatment during the follow-up period of the trial but were informed they should use standard routes to do so. Additional treatments were recorded as a treatment outcome.

### Outcomes

The primary outcome was shoulder pain and function over 12 months after randomisation, measured using the Shoulder Pain and Disability Index (SPADI),^14^ a 13-item measure of patient-reported outcomes in which each item is scored on a 0–10 numerical rating scale (10 being the worst score). Secondary outcomes were SPADI five-item pain subscale and SPADI eight-item function subscale (SPADI subscale items were summed and converted to a 0–100 scale, with higher values denoting more pain or disability); EQ-5D-5L;^15^ fear-avoidance belief questionnaire (physical activity five-item subscale);^16^ pain self-efficacy questionnaire (short form);^17^ insomnia severity index;^18^ participant global impression of change questionnaire;^19^ serious adverse events; return to desired activities, including work, social life, and sport activities; participant exercise adherence; health resource use; additional out-of-pocket expenses; and work absence. Measurements were collected at baseline and then by postal questionnaires at 8 weeks, 6 months, and 12 months after randomisation. Telephone follow-up was used to contact those who did not respond or fully complete the returned questionnaire.

### Statistical analysis

The target sample size was 704 participants, based on 90% power to detect a minimally clinically important between-group difference of 8 points on the SPADI total scale,^20^ assuming a baseline SD of 24·3.^21^ This difference is equivalent to a standardised effect size of 0·33, which required a sample size of 550 participants. NCSS was used to calculate sample size. Allowing for a potential loss to follow-up at 12 months of 20% increased the sample size to 688 participants. We further increased the sample size to 704 participants to take into account the potential for a small clustering effect by physiotherapist (interclass correlation 0·001). The sample size assumed no interaction effect and was powered for the two main-effect comparisons: (1) progressive exercise versus best practice advice to investigate the effects of progressive exercise and (2) subacromial corticosteroid injection versus no injection to investigate the effects of corticosteroid injection. This number of participants provided 80% power and 5% significance to detect an interaction with a standardised effect size of 0·35 or more, if an interaction effect did exist.

All analyses were on an intention-to-treat basis unless specified as otherwise. The presence of an interaction effect was assessed before testing the intervention effects on the primary outcome. The difference in SPADI between intervention groups was estimated over the 12-month period and at each data collection timepoint using a repeated-measures linear mixed-effects regression model adjusted for the fixed-effects age, sex, and baseline SPADI, and random intercepts by centre, physiotherapist, and observations within participant. In the SAP,^11^ we clarified prespecification of the primary outcome measure as being over 12 months, not at 12 months, as described in the protocol.^10^ This prespecification of the primary outcome measure better reflects the planned method of analysis described in the protocol, which used a linear mixed longitudinal model. The data monitoring and ethics committee approved the SAP before the analysis was done and provided a blinded review of the sample size assumptions after 338 participants were recruited and no changes were made to the final sample size. Prespecified subgroup analyses included age (≥65 years), sex, smoking status, increased baseline SPADI score (≥50), and increased baseline pain self-efficacy score (≥8). Secondary outcomes were analysed using the same methods as the primary outcome.

A complier average causal effect (CACE) analysis was used to investigate the role of compliance on the treatment effect. Compliance with intervention was defined in the SAP^11^ for progressive exercise as participants having been signed off for completing treatment or if they received all six physiotherapy sessions and for the injection as receiving the injection.

Statistical significance was set at 1% and corresponding 99% CIs for the primary outcome analyses. For all other outcomes, 5% and 95% CIs were reported. All statistical analyses were done using Stata (version 15). The trial was registered with the International Standard Randomised Controlled Trial Register, ISRCTN16539266, and EuDRACT, 2016-002991-28.

Health economic analyses were done in accordance with the National Institute for Health and Care Excellence reference case.^22^ The analysis was done on an intention-to-treat basis with multiple imputation for missing data. The base-case analysis was done from an NHS and personal social services (PSS) perspective and included the cost over 12 months of delivering each intervention, physiotherapist training, additional medication costs, visits to primary and secondary health-care professionals, outpatient appointments, and hospital stays. Costs were calculated using national UK unit costs expressed in British Pounds Sterling at 2019 prices. Quality-adjusted life-years (QALYs) were estimated from EQ-5D-5L data collected at baseline, 8 weeks, 6 months, and 12 months. Mean costs and mean QALYs were used to derive the incremental cost-effectiveness ratio. Net monetary benefit statistics were calculated as QALYs times willingness to pay threshold minus cost for comparisons between treatment groups. The base-case economic analysis was prespecified as regression analysis with an interaction term adjusting for age, sex and baseline utility. Regression analysis without interaction terms alongside further scenarios was used as a sensitivity analysis to assess whether assuming additive effects changed the conclusions of the analysis.

### Role of the funding source

The funder of the study had no role in study design, data collection, data analysis, data interpretation, or writing of the report.

## Results

**Figure 1:**
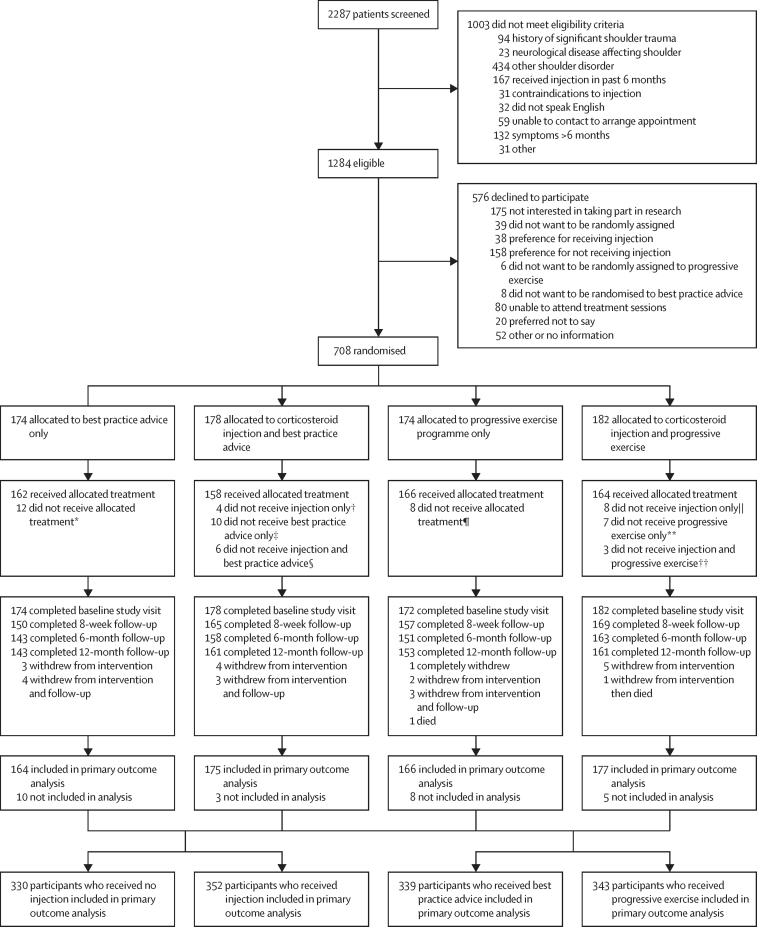
Trial profile All participants with at least one follow-up timepoint SPADI outcome and the baseline variables used in the model were included in the primary outcome analysis. GRASP=Getting it Right: Addressing Shoulder Pain. SPADI=Shoulder Pain and Disability Index. *Reasons for not receiving best practice advice were participant did not attend session (n=5), withdrawal (n=4), or other medical condition (n=3). †Reasons for not receiving injection only were participant declined treatment (n=2) or contraindicated (n=1 taking anticoagulants and n=1 previous reaction to injection). ‡Reasons for not receiving best practice advice only were participant did not attend session (n=5), other medical condition (n=2), received progressive exercise in error (n=3). §Reasons for not receiving injection and best practice advice were participant did not attend session (n=1), participant declined treatment (n=2), other medical condition (n=2), or previous reaction to injection and received non-GRASP treatment (n=1). ¶Reasons for not receiving progressive exercise were participant did not attend session (n=3), received best practice advice in error (n=2), received injection in error (n=1), received non-GRASP treatment (n=1), or withdrawal (n=1). ||Reasons for not receiving injection only were participant declined treatment (n=5) or clinician declined treatment (n=3). **Reasons for not receiving progressive exercise only were received best practice advice in error (n=2), received non-GRASP treatment (n=3), other medical condition (n=1), or participant did not attend session (n=1). ††Reasons for not receiving injection and progressive exercise were participant did not attend session (n=2) or other medical condition (n=1).

Between March 10, 2017, and May 2, 2019, 2287 participants were assessed for eligibility, of whom 1284 (56%) were eligible. 708 (55%) agreed to participate and were randomly assigned to either progressive exercise (n=174 [25%]); best practice advice (n=174 [25%]); progressive exercise preceded by subacromial corticosteroid injection (n=182 [26%]); or best practice advice preceded by subacromial corticosteroid injection (n=178 [25%]; figure 1).

Most participants received treatment as allocated (table 1). Across intervention groups, high protocol adherence was achieved in terms of the delivery, type, and content for the injection, progressive exercise, and best practice advice interventions. 53 physiotherapists delivered corticosteroid injections to 329 (97%) participants and three doctors to ten (3%) participants. Progressive exercise was delivered by 104 physiotherapists to 339 participants and best practice advice was delivered by 83 physiotherapists to 324 participants. Two physiotherapists swapped groups during the trial because of staffing issues and delivered both interventions. We found no difference in attendance rates between those receiving progressive exercise or best practice advice and those who received the intervention in conjunction with corticosteroid injection (table 1). Participants who received the injection were more likely to do their exercises as advised (ie, 5 days per week), compared with those did not receive the injection (appendix p 5).

**Table 1:** Intervention received by treatment group

		Best practice advice (n=174)*	Injection and best practice advice (n=178)*	**Progressive exercise (n=174)**†	**Injection and progressive exercise (n=182)**†
Injection received		..	168 (94%)	..	171 (94%)
Injection not received		..	10 (6%)	..	11 (6%)
	Did not attend	..	1 (<1%)	*..*	2 (1%)
	Participant declined	..	4 (2%)	*..*	4 (2%)
	Clinician declined	..	5 (3%)	*..*	5 (3%)
Received extra injection		..	0	*..*	2 (1%)
Completed exercise treatment‡		162 (93%)	162 (91%)	138 (79%)	139 (76%)
Partial exercise completion§		..	..	29 (17%)	33 (18%)
Received no treatment		12 (7%)	16 (9%)	7 (4%)	10 (5%)
	Did not attend or unable to contact	5 (3%)	6 (3%)	3 (2%)	3 (2%)
	Withdrawal or declined	4 (2%)	2 (1%)	1 (<1%)	0
	Other condition	3 (2%)	4 (2%)	0	2 (1%)
	Received wrong trial intervention	0	3 (2%)	2 (1%)	2 (1%)
	Received non-GRASP treatment	0	1 (<1%)	1 (<%)	3 (2%)
Median number of sessions (IQR)		1 (1–1)	1 (1–1)	4 (3–6)	4 (3–5)
Completed session 1		162 (93%)	162 (91%)	167 (96%)	172 (95%)
Completed session 2		..	..	161 (93%)	160 (88%)
Completed session 3		..	..	144 (83%)	136 (75%)
Completed session 4		..	..	101 (58%)	100 (55%)
Completed session 5		..	..	72 (41%)	69 (38%)
Completed session 6		..	..	44 (25%)	38 (21%)
Participants who received additional sessions		3 (2%)	5 (3%)	3 (2%)	2 (1%)
Additional contact sessions¶		4	6	3	5
	Telephone	2	1	0	1
	Face to face	2	5	3	4

Data are n, n (%), or median (IQR). GRASP=Getting it Right: Addressing Shoulder Pain.

* Maximum one session of best practice advice.

† Up to six sessions of progressive exercise.

‡ For best practice advice, at least one session attended. For progressive exercise, six sessions attended or discharged by clinician because treatment completed (as marked on treatment log), discharged by clinician following patient-initiated follow-up period with no further contact, or referred on for further investigation or treatment.

§ Defined as at least one session attended.

¶ Some participants received more than one additional contact.

Follow-up questionnaires were obtained for 618 (87%) participants at 12 months, 615 (87%) at 6 months, and 641 (91%) at 8 weeks. Overall, 26 (4%) participants withdrew from the trial, 15 from the intervention delivery only and ten from both the intervention and follow-up questionnaire completion (figure 1). Numbers of patient withdrawals were similar across intervention groups (figure 1).

Participant characteristics are shown in table 2. Participants had a mean age of 55·5 years (SD 13·1), 49% were female, 51% were male, and mean symptom duration was 4 months (IQR 3–6; table 2). Intervention groups were well matched in terms of demographic data and clinical and generic health-related quality of life measures (table 2). The overall mean baseline SPADI score was 54·1 (SD 18·5), with higher baseline levels of shoulder pain compared with impairment in shoulder function (table 2).

**Table 2:** Baseline characteristics

		**Best practice advice (n=174)**	**Injection and best practice advice (n=178)**	**Progressive exercise (n=174)**	**Injection and progressive exercise (n=182)**
Age, years		55·9 (13·1)	56·5 (12·4)	54·6 (13·7)	54·8 (13·2)
	18–35*	11 (6%)	13 (7%)	14 (8%)	17 (9%)
	≥36	163 (94%)	165 (93%)	160 (92%)	165 (91%)
Sex*					
	Male	87 (50%)	89 (50%)	90 (52%)	93 (51%)
	Female	87 (50%)	89 (50%)	84 (48%)	89 (49%)
Ethnicity					
	White	160 (92%)	167 (94%)	154 (89%)	167 (92%)
	Other	14 (8%)	11 (6%)	18 (10%)	15 (8%)
	Missing	0	0	2 (1%)	0
Marital status					
	Married or civil union	118 (68%)	107 (60%)	114 (66%)	120 (66%)
	Living with partner	24 (14%)	23 (13%)	22 (13%)	24 (13%)
	Other	32 (18%)	48 (27%)	36 (21%)	38 (21%)
	Missing	0	0	2 (1%)	0
Height, m		1·7 (0·0);n=174	1·7 (0·0);n=178	1·7 (0·2);n=172	1·7 (0·2);n=182
Weight, kg		80·9 (16·6);n=174	82·9 (17·4);n=176	81·2 (18·4);n=170	81·7 (18·0);n=180
Body-mass index, kg/m^2^		27·9 (5·0);n=174	28·7 (5·4);n=176	28·0 (5·4);n=170	28·1 (4·8);n=180
	<18·5	3 (2%)	1 (<1%)	0	0
	18·5 to <25·0	51 (29%)	51 (29%)	50 (29%)	53 (29%)
	25·0 to <30·0	68 (39%)	63 (35%)	73 (42%)	70 (39%)
	≥30·0	52 (30%)	61 (34%)	47 (27%)	57 (31%)
	Missing	0	2 (1%)	4 (2%)	2 (1%)
Smoking status					
	Never smoked	85 (49%)	100 (56%)	99 (57%)	101 (56%)
	Former smoker	66 (38%)	66 (37%)	61 (35%)	63 (35%)
	Current smoker	23 (13%)	12 (7%)	12 (7%)	18 (10%)
	Missing	0	0	2 (1%)	0
Symptom duration, months		4 (2–6);n=173	4 (3–6);n=178	4 (3–6);n=172	4 (3–6);n=182
Affected shoulder					
	Left shoulder	89 (51%)	78 (44%)	83 (48%)	82 (45%)
	Right shoulder	78 (45%)	94 (53%)	84 (48%)	93 (51%)
	Both shoulders	7 (4%)	6 (3%)	5 (3%)	7 (4%)
	Missing	0	0	2 (1%)	0
Hand dominance					
	Left handed	13 (8%)	16 (9%)	21 (12%)	21 (12%)
	Right handed	157 (90%)	153 (86%)	148 (85%)	158 (87%)
	Both	4 (2%)	9 (5%)	3 (2%)	3 (2%)
	Missing	0	0	2 (1%)	0
Current work status					
	Retired	44 (25%)	50 (28%)	40 (23%)	49 (27%)
	Semi-retired	13 (8%)	10 (6%)	9 (5%)	7 (4%)
	Employed	84 (48%)	91 (51%)	98 (56%)	82 (45%)
	Other	33 (19%)	27 (15%)	25 (14%)	43 (24%)
	Missing	0	0	2 (1%)	1 (<1%)
SPADI score					
	Pain subscale	66·0 (17·7); n=174	64·2 (18·3); n=178	60·7 (17·1);n=172	64·6 (17·5);n=182
	Function subscale	48·1 (23·2);n=174	46·3 (22·0);n=178	40·3 (21·0);n=172	42·6 (21·6);n=182
	Overall	57·0 (19·2);n=174	55·3 (18·9);n=178	50·5 (17·5);n=172	53·6 (17·8);n=182
FABQ PA score		15·6 (5·8);n=172	15·7 (5·4);n=177	14·2 (5·5);n=172	14·8 (5·3);n=182
PSEQ-2 score		9·6 (2·4);n=174	9·5 (2·3);n=178	9·8 (2·3);n=172	9·7 (2·3);n=182
ISI score		11·0 (6·7);n=173	10·4 (6·2);n=176	9·5 (5·7);n=170	11·1 (6·4);n=180
RDA score		8·0 (2·7);n=174	8·2 (2·5);n=178	7·6 (2·7);n=172	7·7 (2·6);n=182

Outcomes were collected using patient-reported questionnaires. A participant could choose not to answer a specific question and therefore N might be different for some outcome measures. Data are mean (SD) or n (%). SPADI=Shoulder Pain and Disability Index. FABQ PA=fear-avoidance beliefs questionnaire physical activity. PSEQ-2=pain self-efficacy questionnaire two-item short form. ISI=insomnia severity index. RDA=return to desired activities.

* Stratification factor used in randomisation.

Overall, we found a substantial improvement in SPADI score in each group over 12 months (table 3; appendix p 7). We found no evidence of a statistically significant interaction effect on the primary outcome between progressive exercise and injection over 12 months (interaction coefficient 2·17 [95% CI −2·96 to 7·31]; p=0·41). At 8 weeks the interaction was 3·41 (−3·24 to 10·06; p=0·31), at 6 months −1·88 (−9·98 to 6·22; p=0·65), and at 12 months 2·25 (−5·76 to 10·26; p=0·58). The primary outcome analysis is therefore presented for the two main effect comparisons as prespecified in the SAP (appendix pp 7–8).

**Table 3:** Adjusted and unadjusted mean differences for SPADI score for best practice advice *vs* progressive exercise and injection *vs* no injection

		**Best practice advice**		**Progressive exercise**		**Between-group adjusted difference (99% CI)**	p value*	**No injection**		**Injection**		**Between-group adjusted difference (99% CI)**	p value*
		Unadjusted mean (SD)	Adjusted mean (SE)	Unadjusted mean (SD)	Adjusted mean (SE)			Unadjusted mean (SD)	Adjusted mean (SE)	Unadjusted mean (SD)	Adjusted mean (SE)		
SPADI score													
	Baseline	56·1 (19); n=352	..	52·1 (17·7); n=354	..	..	..	53·8 (18·6); n=346	..	54·4 (18·4); n=360	..	..	..
	8 weeks	38·4 (22·7); n=312	36·96 (1·32); n=297	38·7 (22·9); n=326	39·50 (1·26); n=316	2·54 (−2·16 to 7·23)	0·16	41·7 (22·2); n=306	41·16 (1·24); n=300	35·7 (22·9); n=332	35·52 (1·22); n=313	−5·64 (−9·93 to −1·35)	<0·0001
	6 months	28·2 (23·8); n=301	27·39 (1·33); n=288	25·4 (23·1); n=314	25·87 (1·27); n=307	−1·52 (−6·26 to 3·22)	0·41	26·2 (23·4); n=294	26·33 (1·26); n=289	27·3 (23·7); n=321	26·85 (1·23); n=306	0·52 (−3·82 to 4·86)	0·76
	12 months	24·0 (24·1); n=303	23·67 (1·33); n=288	19·9 (22·7); n=314	20·57 (1·27); n=307	−3·10 (−7·85 to 1·64)	0·092	20·9 (23·1); n=296	21·07 (1·26); n=290	22·8 (23·7); n=321	23·00 (1·23); n=305	1·93 (−2·41 to 6·27)	0·25
	Over 12 months	30·3 (24·3); n=339	29·41 (1·08); n=339	28·1 (24·2); n=343	28·75 (1·03); n=343	−0·66 (−4·52 to 3·20)	0·66	29·7 (24·5); n=330	29·63 (1·0); n=330	28·7 (24·0); n=352	28·53 (0·98); n=352	−1·11 (−4·47 to 2·26)	0·40
CACE estimate		..	..	..	..	−0·27 (−2·69 to 2·16)	0·83	..	..	..	..	−1·50 (−3·61 to 0·61)	0·16

CACE=complier average causal effect. SPADI=Shoulder Pain and Disability Index.

* SPADI analysis adjusted for age, sex, and baseline SPADI score, with random effects within participant, physiotherapist, and centre.

**Figure 2:**
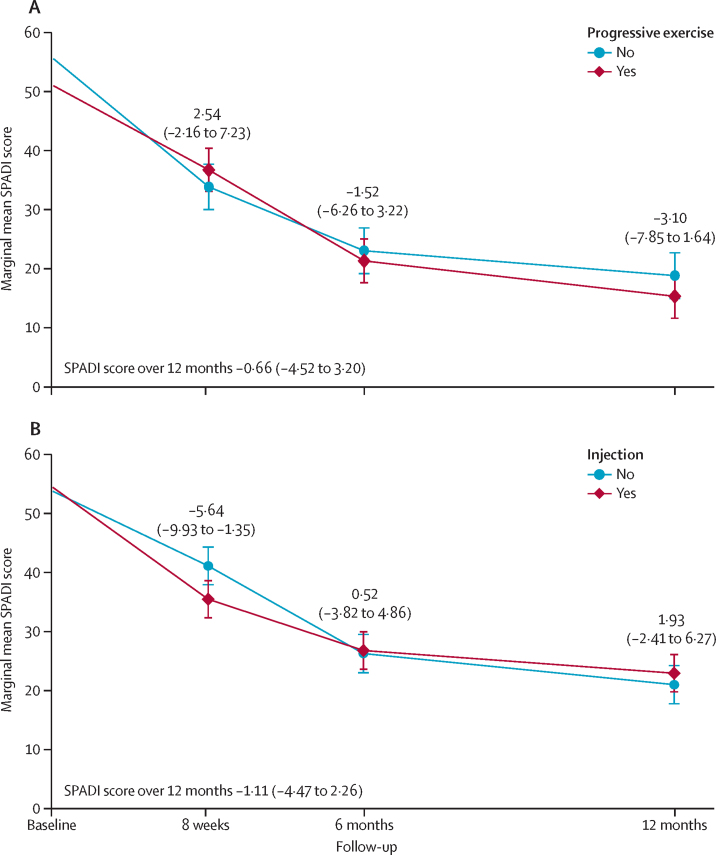
Marginal adjusted mean SPADI scores from baseline to 12 months Results are from the repeated measures mixed-effects model. Figure shows progressive exercise (A) and injection (B). Error bars represent 99% CIs. SPADI=Shoulder Pain and Disability Index.

Over 12 months, we found no evidence of a difference in SPADI score between the progressive exercise intervention and best practice advice (adjusted mean difference −0·66 [99% CI −4·52 to 3·20]; figure 2). We found no difference between progressive exercise and best practice advice at 8 weeks, 6 months, or 12 months (−3·10 [–7·85 to 1·64]; figure 2). Compliance with the progressive exercise intervention did not have a significant effect on the primary outcome (CACE adjusted mean difference −0·27 [95% CI −2·69 to 2·16]; p=0·83; table 3). Over 12 months, we found no evidence of a difference in SPADI scores between patients who received the injection and those who did not (adjusted mean difference −1·11 [–4·47 to 2·26]). We also found no difference between injection and no injection at 6 months and 12 months (1·93 [–2·41 to 6·27; figure 2). We found a small difference in SPADI score at 8 weeks (−5·64 [–9·93 to −1·35]), in favour of injection. Compliance with injection did not have a significant effect on the primary outcome (CACE adjusted mean difference −1·50 [–3·61 to 0·61]; p=0·16; table 3).

Table 4 presents the adjusted analysis results for each of the secondary outcomes (unadjusted results are provided in the appendix [pp 9–10]). We found no evidence of a difference between progressive exercise and best practice advice for secondary outcome measures, other than improvements in patient-reported global impression of treatment for progressive exercise over 12 months, at 6 months, and at 12 months timepoints. We found no evidence of a difference between corticosteroid injection and no injection for secondary outcome measures, except for at 8 weeks, when injection resulted in an improvement in shoulder pain, shoulder function, health-related quality of life, sleep disturbance, return to desired activities, and global impression of treatment (table 4).

**Table 4:** Adjusted mean differences for secondary outcomes for best practice advice *vs* progressive exercise and injection *vs* no injection

	**Best practice advice**	**Progressive exercise**	**Between-group adjusted difference (95% CI)**	p value*	**No injection**	**Injection**	**Between-group adjusted difference (95% CI)**	p value*
	Adjusted mean (SE)	Adjusted mean (SE)			Adjusted mean (SD)	Adjusted mean (SE)		
**SPADI pain subscale score**								
8 weeks	49·9 (1·4); n=300	25·9 (1·3); n=316	2·12 (−1·94 to 6·17)	0·31	49·9 (1·4); n=300	42·9 (1·4); n=315	−7·38 (−11·10 to −3·67)	<0·0001
6 months	32·0 (1·4); n=289	20·6 (1·3); n=307	−3·06 (−7·16 to 1·04)	0·14	32·0 (1·4); n=289	32·9 (1·4); n=306	0·89 (−2·88 to 4·66)	0·64
12 months	25·8 (1·4); n=290	39·5 (1·3); n=307	−4·01 (−8·11 to 0·09)	0·055	25·8 (1·4); n=290	27·8 (1·4); n=306	2·05 (−1·72 to 5·81)	0·29
Over 12 months	36·0 (1·3); n=339	34·5 (1·2); n=343	−1·61 (−4·94 to 1·72)	0·34	36·0 (1·3); n=339	34·5 (1·1); n=343	−1·55 (−4·46 to 1·37)	0·30
**SPADI function subscale score**								
8 weeks	29·40 (1·24); n=298	31·77 (1·19); n=316	2·37 (−1·01 to 5·76)	0·17	32·59 (1·18); n=301	28·75 (1·16); n=313	−3·84 (−6·95 to −0·73)	0·015
6 months	20·95 (1·26); n=288	20·65 (1·21); n=307	−0·30 (−3·72 to 3·12)	0·86	20·69 (1·19); n=289	20·89 (1·17); n=306	0·20 (−2·95 to 3·35)	0·90
12 months	18·74 (1·26); n=288	16·12 (1·20); n=307	−2·61 (−6·03 to 0·81)	0·13	16·38 (1·19); n=290	18·34 (1·17); n=305	1·97 (−1·18 to 5·11)	0·22
Over 12 months	23·1 (1·0); n=339	22·9 (1·0); n=343	−0·15 (−2·92 to 2·61)	0·91	23·31 (0·93); n=339	22·72 (0·92); n=343	−0·59 (−3·02 to 1·83)	0·63
**EQ-5D-5L**								
8 weeks	0·71 (0·01); n=274	0·69 (0·01); n=303	−0·02 (−0·05 to 0·00)	0·091	0·69 (0·01); n=278	0·71 (0·01); n=299	0·03 (0·00 to 0·05)	0·045
6 months	0·75 (0·01); n=273	0·74 (0·01); n=291	−0·00 (−0·03 to 0·02)	0·74	0·75 (0·01); n=273	0·74 (0·01); n=291	−0·01 (−0·03 to 0·02)	0·71
12 months	0·77 (0·01); n=281	0·78 (0·01); n=290	0·01 (−0·02 to 0·04)	0·51	0·78 (0·01); n=276	0·76 (0·01); n=295	−0·02 (−0·05 to 0·01)	0·16
Over 12 months	0·74 (0·01); n=330	0·74 (0·01); n=332	−0·01 (−0·03 to 0·02)	0·57	0·74 (0·01); n=319	0·74 (0·01); n=343	0·00 (−0·02 to 0·02)	0·92
**Fear-avoidance belief questionnaire (physical activity)**								
8 weeks	11·62 (0·37); n=269	11·94 (0·35); n=301	0·32 (−0·68 to 1·33)	0·53	12·02 (0·36); n=274	11·59 (0·35); n=296	−0·43 (−1·39 to 0·53)	0·38
6 months	9·93 (0·37); n=266	9·51 (0·36); n=281	−0·42 (−1·44 to 0·60)	0·42	9·70 (0·37); n=262	9·72 (0·35); n=285	0·03 (−0·95 to 1·01)	0·95
12 months	9·29 (0·37); n=266	8·32 (0·36); n=284	−0·97 (−1·99 to 0·05)	0·061	8·55 (0·37); n=265	9·01 (0·35); n=285	0·47 (−0·51 to 1·44)	0·35
Over 12 months	10·3 (0·30); n=324	9·94 (0·28); n=332	−0·35 (−1·16 to 0·46)	0·40	8·22 (0·10); n=316	8·41 (0·10); n=340	0·02 (−0·74 to 0·77)	0·97
**Pain self-efficacy questionnaire**								
8 weeks	10·25 (0·13); n=271	10·19 (0·12); n=300	−0·06 (−0·39 to 0·27)	0·74	10·13 (0·13); n=275	10·30 (0·12); n=296	0·17 (−0·16 to 0·50)	0·31
6 months	10·41 (0·13); n=267	10·42 (0·13); n=282	0·01 (−0·33 to 0·34)	0·96	10·44 (0·13); n=264	10·39 (0·13); n=285	−0·05 (−0·38 to 0·28)	0·77
12 months	10·59 (0·13); n=267	10·70 (0·13); n=284	0·12 (−0·22 to 0·45)	0·49	10·80 (0·13); n=266	10·50 (0·13); n=285	−0·30 (−0·63 to 0·03)	0·078
Over 12 months	10·41 (0·10); n=325	10·43 (0·10); n=332	0·02 (−0·23 to 0·27)	0·86	10·45 (0·10); n=317	10·40 (0·10); n=340	−0·06 (−0·31 to 0·19)	0·66
**Insomnia severity index**								
8 weeks	7·46 (0·32); n=267	8·09 (0·31); n=294	0·63 (−0·25 to 1·50)	0·16	8·57 (0·31); n=270	7·07 (0·30); n=291	−1·50 (−2·32 to −0·68)	0·0003
6 months	6·20 (0·32); n=264	6·20 (0·31); n=281	−0·01 (−0·89 to 0·88)	0·99	6·21 (0·31); n=262	6·18 (0·30); n=283	−0·03 (−0·86 to 0·80)	0·95
12 months	5·92 (0·32); n=267	5·40 (0·31); n=282	−0·52 (−1·40 to 0·36)	0·25	5·48 (0·31); n=265	5·80 (0·30); n=284	0·32 (−0·51 to 1·15)	0·45
Over 12 months	6·53 (0·27); n=323	6·57 (0·26); n=329	0·04 (−0·69 to 0·77)	0·92	6·77 (0·25); n=314	6·35 (0·25); n=338	−0·41 (−1·08 to 0·26)	0·23
**Return to desired activities**								
8 weeks	6·08 (0·14); n=270	6·33 (0·13); n=297	0·25 (−0·12 to 0·62)	0·19	6·49 (0·14); n=273	5·96 (0·13); n=294	−0·53 (−0·89 to −0·17)	0·0042
6 months	5·42 (0·14); n=267	5·10 (0·14); n=284	−0·31 (−0·69 to 0·06)	0·10	5·27 (0·14); n=266	5·24 (0·14); n=285	−0·03 (−0·39 to 0·34)	0·89
12 months	4·81 (0·14); n=268	4·67 (0·14); n=285	−0·14 (−0·51 to 0·24)	0·47	4·63 (0·14); n=268	4·84 (0·14); n=285	0·21 (−0·15 to 0·57)	0·26
Over 12 months	5·44 (0·11); n=325	5·38 (0·11); n=332	−0·06 (−0·36 to 0·23)	0·66	5·47 (0·11); n=317	5·35 (0·11); n=340	−0·12 (−0·40 to 0·16)	0·41
**Global impression of treatment**								
8 weeks	7·65 (0·13); n=269	7·75 (0·13); n=298	0·11 (−0·25 to 0·47)	0·56	7·34 (0·13); n=273	8·03 (0·13); n=294	0·69 (0·35 to 1·03)	<0·0001
6 months	8·16 (0·13); n=267	8·69 (0·13); n=285	0·53 (0·17 to 0·90)	0·0038	8·42 (0·13); n=267	8·45 (0·13); n=285	0·04 (−0·31 to 0·39)	0·83
12 months	8·57 (0·13); n=270	9·08 (0·13); n=286	0·51 (0·14 to 0·87)	0·0060	8·91 (0·13); n=269	8·76 (0·13); n=287	−0·14 (−0·49 to 0·20)	0·42
Over 12 months	8·12 (0·10); n=326	8·50 (0·10); n=332	0·38 (0·10 to 0·66)	0·0074	8·22 (0·10); n=317	8·41 (0·10); n=347	0·20 (−0·06 to 0·46)	0·14

SPADI=shoulder pain and disability index.

* Outcome analysis adjusted for age, sex, and baseline outcome value, with random effects within participant, physiotherapist, and centre.

We found no difference in prespecified subgroup analyses over 12 months for the primary outcome, with the exception of corticosteroid injection, in which the effect was stronger at 8 weeks in participants with a higher baseline SPADI score (adjusted MD −9·67 [99% CI −15·37 to −3·97]), compared with those who received the injection but had a lower baseline SPADI score (−0·36 [–6·87 to 6·16]; appendix pp 13–18). No serious adverse events were reported. Three participants reported undergoing surgery to repair rotator cuff tear (n=1; injection plus progressive exercise), frozen shoulder that developed after randomisation (n=1; best practice advice), and subacromial decompression and repair of rotator cuff tear (n=1; injection plus best practice advice).

The base-case cost-effectiveness analysis showed that over the 12-month period, participants in the best practice group accrued on average 0·737 QALYs (95% CI 0·710–0·763) and an NHS cost of £195 per patient (appendix p 11). Adding progressive exercise to best practice advice gained an additional 0·019 QALYs (p=0·22) at an additional NHS and PSS cost of £52 per patient (p=0·25; appendix p 11). Adding corticosteroid injection to best practice advice gained 0·021 QALYs (p=0·18) and increased the total NHS and PSS cost by £10 per patient (p=0·75). At a ceiling ratio of £20 000 per QALY, best practice advice plus injection was found to have a 54·93% probability of being best value for money (appendix p 19). Best practice advice plus injection cost £476 per QALY gained compared with best practice advice alone. This combination of treatments was more cost-effective than progressive exercise alone and progressive exercise plus injection, being non-significantly less costly and accruing more QALYs. Sensitivity analyses assuming additive effects, taking a societal perspective, and varying the cost of training physiotherapists confirmed the base-case conclusion that best practice advice plus injection is expected to be the best value for money in the UK, at a ceiling ratio of £20 000 per QALY.^23^

## Discussion

To our knowledge, the GRASP trial is the largest randomised controlled trial to date that has investigated the effects of exercise interventions and corticosteroid injections in adults with a new episode of shoulder pain attributable to a rotator cuff disorder. Our findings showed no difference in the primary outcome (SPADI score) or other prespecified secondary outcomes between participants randomly assigned to receive either progressive exercise compared with best practice advice, or subacromial corticosteroid injection compared with no injection, when analysed over 12 months. We found some evidence that corticosteroid injection improves shoulder pain and function at 8 weeks. The greatest benefit was in the subgroup of participants who reported high SPADI scores at baseline but because this finding was based on subgroup analysis it should be viewed with caution. Irrespective of allocated intervention, participants' shoulder pain and function improved over time, although SPADI scores at 12 months showed that the condition did not resolve completely. The most cost-effective intervention in the UK was the best practice advice session with a physiotherapist plus corticosteroid injection, although substantial uncertainty exists around this conclusion. Although the benefits of corticosteroid injection were limited both in size and to the early phase of recovery this combination of interventions was best in terms of their cost-effectiveness.

A Cochrane review published in 2016 highlighted insufficient evidence about the long-term effects of treatments offered by physiotherapists for rotator cuff disorders.^4^ In our own systematic review we identified seven trials^24^, ^25^, ^26^, ^27^, ^28^, ^29^, ^30^ between 2013 and 2020, comparing the effects of supervised versus unsupervised exercise, or no intervention, in people with a rotator cuff disorder. All the trials were small, apart from two moderate sized trials with 271 and 256 participants each,^28^, ^30^ and only two^26^, ^30^ reported on the effect of exercise on shoulder pain and function at 12 months, four reported medium-term follow-up data (4–6 months), and the remaining three reported outcomes at 6 weeks or less. Most studies concluded little or no difference between supervised and unsupervised exercise at 6 months and 12 months, with the exception of the SUPPORT trial,^30^ which showed a benefit of supervised exercise compared with a simple exercise leaflet at 6 months but not at 12 months.

Despite widespread use of corticosteroid injection for treating tendinopathy,^9^ we only identified one small trial^31^ comparing the effects of corticosteroid injection versus no injection in people with a rotator cuff disorder. This trial found no difference in shoulder pain and function when analysed at 3 months or 6 months. An additional ten trials compared the effects of corticosteroid injection versus placebo injection; three assessed shoulder pain and function showing benefit of injection in the short term (≤8 weeks) but not medium term (3–6 months; unpublished). No trials provided data beyond 6-month follow-up and none reported any serious adverse events as a result of injection. Poor response from injection has been attributed to inaccurate placement of the injection.^30^ However, high quality evidence from the SUPPORT trial shows that ultrasound guidance conferred no additional benefit over unguided corticosteroid injection.^30^ These findings reinforce the importance of the GRASP trial findings in terms of their definitive nature, length of follow-up, and short-term benefit of corticosteroid injection.

The strength of the GRASP trial, as a definitive, multicentre, factorial, randomised controlled trial, is that in the absence of any significant interaction we were able to assess the effects of our two main intervention comparisons. We recruited 708 participants and had a lower-than-estimated loss-to-follow-up rate of 13% at 12 months, so the trial was adequately powered to detect a statistically and clinically important difference between interventions should one have been present. Participants were recruited from 20 NHS trusts in the UK, which in terms of location and size make the sample fairly representative of NHS patients as a whole. Physiotherapists were trained to deliver either best practice advice or progressive exercise to minimise possible contamination between treatment groups. Only two physiotherapists swapped treatment groups during the trial and delivered both interventions. Despite some initial concerns from physiotherapists raised during site training regarding the adequacy of a single session to start the self-guided best practice advice exercise programme, very few participants required an additional contact session during the trial.

A limitation was that we could not mask participants and treating physiotherapists because of the nature of the interventions being tested, reflecting a difficulty that is common to pragmatic rehabilitation trials. This lack of masking reflected in the open-label design has the potential to overestimate as opposed to underestimate the treatment effect, thus strengthening our findings, which showed no difference between treatment interventions over 12 months. When practical, members of the trial team were masked until after data analysis was complete, with the exception of the data entry personnel who entered data from anonymised questionnaires, which included some details on treatments received. Data entry was checked as part of quality assurance processes and any bias associated with this process was considered minimal. Additionally, a masked analysis of data was undertaken before the final data lock.

Early and effective management of rotator cuff disorders is important given evidence from the CSAW (Can Shoulder Arthroscopy Work) trial^32^ and updated Cochrane review^33^ showing no benefit from subacromial decompression surgery. Subacromial corticosteroid injection, although providing modest short-term benefit, was associated with participants being more likely to report doing their exercises as advised (ie, 5 days per week) in both the progressive exercise and best practice advice intervention groups. Physiotherapists delivering the best practice advice focused on strategies to promote self-management and independent progression of exercise, adherence to exercise, and addressing barriers to exercise. The exercises prescribed were those within the range that physiotherapists deliver in usual practice. As a result, we believe the implementation of the best practice advice intervention into the NHS would be straightforward and involve somewhat small training costs. For some patients, a single session might not be appropriate—eg, those with low levels of literacy or inability to engage with self-management care, in which case additional physiotherapy sessions might be required.

Our population was predominantly White British, with the proportion of such participants higher than that of the population in England as a whole.^34^ The prevalence of rotator cuff disorder in ethnic minority groups is not well known or understood and so inferring what influence this over-representation might have on the generalisability of our results is difficult. Some participants reported ongoing pain and impaired shoulder function at 12 months; thus future research is needed to better understand the natural history of rotator cuff disorders, including whether symptoms resolve over an extended time period. Very few participants reported undergoing surgery and extended follow-up would also address concerns regarding later surgery and possible long-term harm after corticosteroid injection and surgery's potential effects on tendon structure.

In conclusion, the GRASP trial showed that progressive exercise was not superior to a best practice advice session with a physiotherapist. Subacromial corticosteroid injection improved shoulder pain and function and provided modest short-term benefit. Best practice advice in combination with corticosteroid injection is expected to be the most cost-effective treatment combination for use of NHS resources, although this conclusion is uncertain.

## Data sharing

All data requests should be submitted to the corresponding author (SH) for consideration as agreed in our publication plan. Access to anonymised data may be granted following review with the trial management group and agreement of the co-chief investigators (SH and SEL).

## Declaration of interests

SH is a member of the UK National Institute for Health Research (NIHR) Health Technology Assessment (HTA) Clinical Evaluation and Trials Committee (Nov 1, 2018, to Nov 30, 2022). DJK holds an NIHR postdoctoral fellowship. IRM holds an NIHR pre-doctoral fellowship. SJD reports grants from the NIHR HTA programme during the conduct of the study. HD is partly funded by an NIHR senior research fellowship through the Biomedical Research Centre, Oxford, UK. AC is a member of the UK Research and Innovation/Medical Research Council Developmental Pathway Funding Scheme panel. He was chief investigator of the NIHR HTA UK Rotator Cuff Surgery (UKCUFF) trial and the CSAW trial. He is a consultant to the Novartis musculoskeletal board. He is chief investigator of an NIHR i4i trial of a novel electrospun patch and of a Wellcome Trust-funded trial of a novel electrospun suture in rotator cuff repair surgery. He was director of the NIHR Musculoskeletal British Research Unit from 2008 to 2017 and is musculoskeletal theme lead for the NIHR Oxford comprehensive British Research Centre. ZH reports grants from the NIHR HTA programme during the conduct of the study and is paid personal fees by various health-care trusts and individuals to train health-care professionals in cognitive behavioural approaches, outside the submitted work. AJ is a council member of the British Shoulder and Elbow Society. She is co-applicant for the NIHR HTA (Partial Rotator Cuff Tear Repair (PROCURE) trial. CL is chair of the Chartered Society of Physiotherapy scientific panel. He is chief investigator of the NIHR post-doctoral fellowship-funded Surgery versus PhysiothErapist-leD exercise (SPeEDy) study. He was previously lead researcher for the NIHR Research for Patient Benefit funded Rehabilitation Following Rotator Cuff Repair (RaCeR) study and chief investigator for the NIHR doctoral fellowship-funded SELF (a self-managed single exercise programme versus usual physiotherapy treatment for rotator cuff tendinopathy) study. SEL reports grants from the NIHR HTA programme during the conduct of the study and was a member of the following boards: HTA Additional Capacity Funding Board 2012–15; HTA Clinical Trials Board 2010–15; HTA End of Life Care and Add on Studies 2015–15; HTA Funding Boards Policy Group (formerly CSG) 2010–15; HTA Maternal, Neonatal and Child Health Methods Group 2013–15; HTA post-board funding teleconference (Prioritisation Group members to attend) 2010–15; HTA Primary Care Themed Call board 2013–14; HTA Prioritisation Group 2010–15; and NIHR Clinical Trials Unit Standing Advisory Committee 2012–16. All other authors declare no completing interests.
