# Supplementary material for: Progressive exercise compared with best practice advice, with or without corticosteroid injection, for the treatment of patients with rotator cuff disorders (GRASP): a multicentre, pragmatic, 2 × 2 factorial, randomised controlled trial
Source: Lancet. 2021 Jul 31;398(10298):416–28. doi: 10.1016/S0140-6736(21)00846-1 (PMC8343092; doi:10.1016/S0140-6736(21)00846-1)
Supplement: Supplementary appendix [file mmc1.pdf]

THE LANCET

Supplementary appendix

This appendix formed part of the original submission and has been peer reviewed. We post it as supplied by the authors.

Supplement to: Hopewell S, Keene DJ, Marian IR, et al. Progressive exercise compared with best practice advice, with or without corticosteroid injection, for the treatment of patients with rotator cuff disorders (GRASP): a multicentre, pragmatic, 2 × 2 factorial, randomised controlled trial. *Lancet* 2021; published online July 12. http://dx.doi.org/10.1016/S0140-6736(21)00846-1.

APPENDIX: ADDITIONAL TABLES AND FIGURES

Characteristics of participating physiotherapists

Agenda for Change Grade	Band 5	Band 6	Band 7	Band 8a	Other[a]	Total[b]
Injectors, n (%)	0 (0)	6 (11)	13 (23)	34 (61)	3 (5)	56 (100)
Best Practice Advice, n (%)	17 (20)	38 (46)	22 (27)	6 (7)	0 (0)	83 (100)
Progressive Exercise, n (%)	16 (15)	55 (53)	28 (27)	5 (5)	0 (0)	104 (100)

[a] 2 orthopaedic consultants and 1 specialist registrar in orthopaedics

[b] some physiotherapists provided both injection and exercise interventions; 2 physiotherapists delivered both exercise interventions

Content of sub-acromial corticosteroid injection

Content of injection[a]	Injection + Progressive Exercise N (received injection)=171	Injection + Best Practice Advice N (received injection)=168	Total N (received injection)=339
	**N (%), median dose/volume (IQR)**	**N (%), median dose/volume (IQR)**	**N (%), median dose/volume (IQR)**
**Corticosteroid**			
**Methylprednisolone, mg**	60 (35%), 40 (32-40)	61 (36%), 40 (30-30)	121 (36%),40 (30-40)
**Triamcinolone acetonide, mg**	111 (65%), 40 (20-20)	107 (64%), 40 (20-40)	218 (64%), 40 (20-40)
**Local anaesthetic**			
**1% lidocaine, mls**	148 (87%), 4 (2-5)	146 (87%), 4 (2-5)	294 (87%), 4 (2-5)
**0.5% Bupivacaine HCl, mls**	18 (11%), 9 (9-9)	15 (9%), 9 (9-10)	33 (10%), 9 (9-9)
**None**	5 (3%)	7 (4%)	12 (4%)
**Shoulder**			
**Right shoulder**	91 (53%)	90 (54%)	181 (53%)
**Left shoulder**	80 (47%)	78 (46%)	158 (47%)
**Injection site**			
**Anterior subacromial**	13 (7%)	14 (8%)	27 (8%)
**Posterior subacromial**	142 (83%)	136 (81%)	278 (82%)
**Lateral subacromial**	15 (9%)	15 (9%)	30 (9%)
**Glenohumeral joint**	1 (1%)	3 (2%)	4 (1%)

[a] % of participants that received injection

CONTENT OF BEST PRACTICE ADVICE INTERVENTION

Treatment components, N (%)[a]		Best Practice Advice only N (received BPA)=162	Injection + Best Practice Advice N (received BPA)=162	Total N (received BPA)=324
**Exercises prescribed**		162 (100)	162 (100)	324 (100)
**Advice / information booklet provided**		161 (99)	162 (100)	323 (100)
**Exercise diary issued**		162 (100)	159 (98)	321 (99)
**Action planner completed**		160 (99)	160 (99)	320 (99)
**Exercise video online / DVD provided**		160 (99)	158 (98)	318 (98)
**Exercises[b]**				
**1**	a) Shoulder abduction supported by table in sitting	31 (10)	20 (6)	51 (8)
	b) Shoulder flexion supported by table in sitting	22 (7)	18 (6)	40 (6)
**2**	a) Isometric shoulder external rotation	29 (9)	30 (9)	59 (9)
	b) Isometric shoulder abduction	36 (11)	35 (11)	71 (11)
**3**	a) Resisted shoulder external rotation - 0° [c]	89 (28)	82 (26)	171 (27)
	b) Shoulder flexion up a wall	60 (19)	66 (21)	126 (20)
**4**	a) Resisted shoulder external rotation - 90° [c]	16 (5)	32 (10)	48 (8)
	b) Shoulder raise using a weight	26 (8)	30 (9)	56 (9)
**5**	a) Resisted shoulder abduction/external rotation[c]	11 (3)	7 (2)	18 (3)
**Total**		320 (100)	320 (100)	640 (100)

[a]% of those who attended best practice advice

[b]Number of times exercise prescribed; number of participants attending = 324 but participants usually received more than one exercise

[c] Using resistance band

Content of progressive exercise Intervention

Session[a]	Intervention	Attended	Exercises prescribed	Advice / information booklet provided	Exercise diary issued / reviewed	Action planner completed / reviewed	Confidence ruler reviewed
		N (%)	N (%)	N (%)	N (%)	N (%)	N (%)
**1**	Progressive Exercise only	167 (100)	167 (100)	166 (99)	164 (98)	163 (98)	165 (99)
	Injection + Progressive Exercise	172 (100)	172 (100)	171 (99)	171 (99)	172 (100)	172 (100)
**2**	Progressive Exercise only	161 (100)	160 (99)	30 (19)	153 (95)	91 (57)	150 (93)
	Injection + Progressive Exercise	160 (100)	158 (99)	28 (18)	147 (92)	99 (62)	144 (90)
**3**	Progressive Exercise only	144 (100)	138 (96)	23 (16)	133 (92)	88 (61)	124 (86)
	Injection + Progressive Exercise	136 (100)	132 (97)	25 (18)	126 (93)	88 (65)	117 (86)
**4**	Progressive Exercise only	101 (100)	96 (95)	22 (22)	94 (93)	69 (68)	88 (87)
	Injection + Progressive Exercise	100 (100)	94 (94)	19 (19)	93 (93)	67 (67)	88 (88)
**5**	Progressive Exercise only	72 (100)	66 (92)	16 (22)	66 (92)	52 (72)	62 (86)
	Injection + Progressive Exercise	69 (100)	66 (96)	11 (16)	64 (93)	49 (71)	62 (90)
**6**	Progressive Exercise only	44 (100)	42 (95)	14 (32)	38 (86)	31 (70)	37 (84)
	Injection + Progressive Exercise	38 (100)	36 (95)	4 (11)	35 (92)	25 (66)	34 (89)

[a] % of those who attended session

PARTICIPANT REPORTED EXERCISE ADHERENCE

	Best Practice Advice		Progressive Exercise		No Injection		Injection		Overall	
	n	%	n	%	n	%	n	%	n	%
**8 weeks**										
Every day	34	9·7%	40	11·2%	34	9·8%	40	11·1%	74	10·5%
6 days per week	15	4·3%	15	4·2%	15	4·3%	15	4·2%	30	4·2%
5 days per week	152	43·2%	215	60·4%	160	46·0%	207	57·5%	367	51·8%
4 days per week	26	7·4%	19	5·3%	27	7·8%	18	5·0%	45	6·4%
3 days per week	15	4·3%	6	1·7%	15	4·3%	6	1·7%	21	3·0%
2 days per week	9	2·6%	2	0·6%	6	1·7%	5	1·4%	11	1·6%
1 day per week	3	0·9%	0	0·0%	1	0·3%	2	0·6%	3	0·4%
None	28	8·0%	11	3·1%	18	5·2%	21	5·8%	39	5·5%
Missing	70	19·9%	48	13·5%	72	20·7%	46	12·8%	118	16·7%
**6 months**										
Every day	19	5·4%	14	3·9%	14	4·0%	19	5·3%	33	4·7%
6 days per week	5	1·4%	4	1·1%	7	2·0%	2	0·6%	9	1·3%
5 days per week	53	15·1%	66	18·5%	42	12·1%	77	21·4%	119	16·8%
4 days per week	24	6·8%	26	7·3%	22	6·3%	28	7·8%	50	7·1%
3 days per week	33	9·4%	33	9·3%	34	9·8%	32	8·9%	66	9·3%
2 days per week	27	7·7%	19	5·3%	20	5·7%	26	7·2%	46	6·5%
1 day per week	13	3·7%	11	3·1%	13	3·7%	11	3·1%	24	3·4%
None	101	28·7%	117	32·9%	116	33·3%	102	28·3%	218	30·8%
Missing	76	21·6%	65	18·3%	79	22·7%	62	17·2%	141	19·9%
**12 months**										
Every day	15	4·3%	16	4·5%	9	2·6%	22	6·1%	31	4·4%
6 days per week	3	0·9%	1	0·3%	3	0·9%	1	0·3%	4	0·6%
5 days per week	22	6·3%	15	4·2%	13	3·7%	24	6·7%	37	5·2%
4 days per week	16	4·5%	12	3·4%	14	4·0%	14	3·9%	28	4·0%
3 days per week	24	6·8%	26	7·3%	21	6·0%	29	8·1%	50	7·1%
2 days per week	19	5·4%	22	6·2%	17	4·9%	24	6·7%	41	5·8%

1 day per week	18	5·1%	16	4·5%	21	6·0%	13	3·6%	34	4·8%
None	165	46·9%	183	51·4%	176	50·6%	172	47·8%	348	49·2%
Missing	69	19·6%	64	18·0%	74	21·3%	59	16·4%	133	18·8%

Progressive exercise versus best practice advice and injection versus best practice advice inside-the-table analysis of SPADI overall

SPADI over 12 months	Progressive Exercise vs Best Practice Advice		Injection + Best Practice Advice vs Best Practice Advice	
	N=174	N=174	N=178	N=174
**Unadjusted mean (SD)[a]**	27·36 (23·49), 166	32·21 (25·39), 164	28·55 (23·11), 175	32·21 (25·39), 164
**Adjusted mean (SE)[b]**	28·79 (1·39), 154	30·55, (1·43), 146	28·32, (1·41), 151	30·55, (1·43), 146
**Unadjusted difference, (99%CI)[a]**	-4·64 (-10·20, 0·91)		-3·50 (-8·99, 1·99)	
**Adjusted difference, (99%CI), p-value[b]**	-1·76 (-6·91, 3·39), 0·380		-2·23 (-7·03, 2·57), 0·230	

[a]SPADI unadjusted analysis using a mixed effects model with random effects within participant with time-by-treatment interaction, 1,870 participant data points contribute to the unadjusted model

[b]SPADI adjusted analysis using a mixed effects model with fixed effects for age, sex, baseline SPADI and random effects for observations within participant, physiotherapist and centre, with time-by-treatment interaction, 1,869 participant data points contribute to the adjusted model

SD-Standard Deviation; SE-Standard Error; CI-Confidence Interval

Progressive exercise versus best practice advice and injection versus best practice advice inside-the-table analysis of SPADI at each time point

SPADI at each time point	Progressive Exercise vs Best Practice Advice				Injection vs Best Practice Advice			
	Adjusted mean (SE), N	Adjusted mean (SE), N	Adjusted difference (99%CI)[a]	p-value	Adjusted mean (SE), N	Adjusted mean (SE), N	Adjusted difference (99%CI)[a]	p-value
8 weeks	41·22(1·78), 156	41·09(1·72), 149	-0·13 (-6·52, 6·27)	0·959	32·89(1·75), 163	37·97(1·68), 149	-8·33 (-14·46, -2·19)	0·000
6 months	26·99(1·81), 151	25·71(1·74), 143	-1·28 (-7·76, 5·20)	0·611	27·75(1·76), 158	26·02(1·70), 143	0·76 (-5·45, 6·97)	0·752
12 months	23·12(1·81), 153	19·19(1·74), 143	-3·93 (-10·40, 2·55)	0·118	24·17(1·76), 160	21·90(1·71,) 143	1·05 (-5·15, 7·26)	0·663

[a]SPADI adjusted analysis using a mixed effects model with fixed effects for age, sex, baseline SPADI and random effects for observations within participant, physiotherapist and centre, with time-by-treatment interaction, 1,869 participant data points contribute to the adjusted model
SE-Standard Error; CI-Confidence Interval

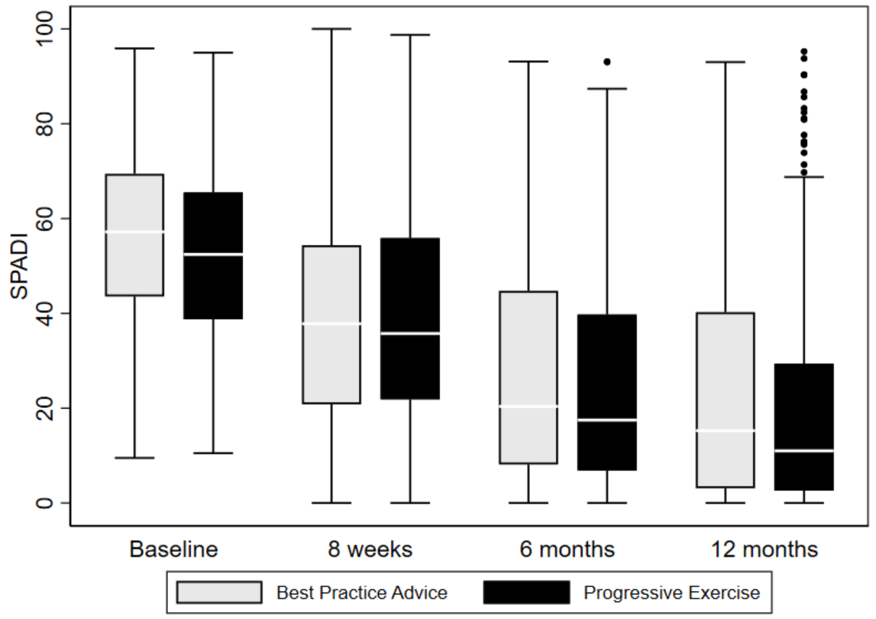

Box and whisker plots for raw SPADI scores for Progressive Exercise vs Best Practice Advice from baseline to 12 months. SPADI range is from 0 to 100, higher scores are indicative of more disability

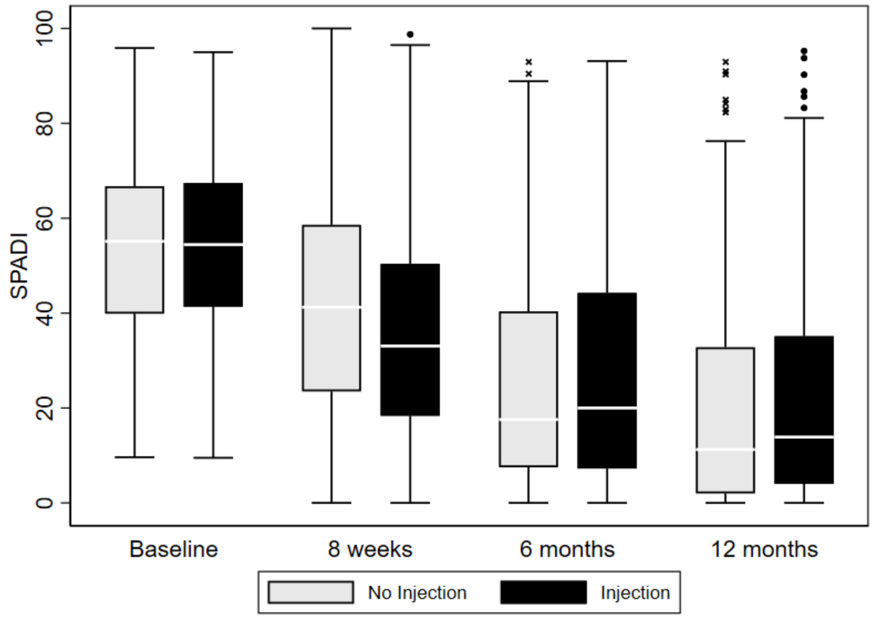

Box and whisker plots for raw SPADI scores for Injection vs No Injection from baseline to 12 months. SPADI range is from 0 to 100, higher scores are indicative of more disability

Secondary outcomes analysis: unadjusted mean and standard deviation

	Best Practice Advice		Progressive Exercise		No Injection		Injection	
	N	Unadjusted mean (SD)	N	Unadjusted mean (SD)	N	Unadjusted mean (SD)	N	Unadjusted mean (SD)
**SPADI Pain**								
Baseline	352	65·1 (18)	354	62·7 (17·4)	346	63·3 (17·6)	360	64·4 (17·9)
8 weeks	314	46·0 (24·5)	326	46·7 (24·5)	306	50·2 (23·4)	334	42·7 (24·9)
6 months	301	34·4 (26·4)	314	30·9 (25·7)	294	31·7 (25·6)	321	33·5 (26·5)
12 months	304	28·7 (27·1)	314	24·6 (25·5)	296	25·5 (25·9)	322	27·7 (26·8)
Over 12 months	339	36·6 (27·0)	343	34·2 (26·9)	339	35·9 (27·1)	343	34·7 (26·8)
**SPADI Function**								
Baseline	352	47·2 (22·6)	354	41·5 (21·3)	346	44·2 (22·4)	360	44·5 (21·9)
8 weeks	313	31·3 (23·6)	326	30·8 (23·5)	307	33·3 (23·5)	332	29·0 (23·3)
6 months	301	22·0 (22·6)	314	19·9 (22·1)	294	20·7 (22·6)	321	21·2 (22·2)
12 months	303	19·4 (22·6)	314	15·1 (21)	296	16·3 (21·8)	321	18·0 (22·1)
Over 12 months	339	24·3 (23·5)	343	22·0 (23·2)	339	23·6 (23·7)	343	22·8 (23·0)
**EuroQol EQ-5D-5L**								
Baseline	352	0.64 (0.20)	353	0.66 (0.20)	346	0.67 (0.18)	359	0.64 (0.18)
8 weeks	283	0.71 (0.18)	311	0.70 (0.17)	279	0.69 (0.17)	315	0.71 (0.18)
6 months	285	0.75 (0.18)	298	0.75 (0.20)	277	0.76 (0.18)	306	0.74 (0.20)

12 months	296	0.77 (0.18)	298	0.78 (0.20)	282	0.79 (0.18)	312	0.76 (0.19)
Over 12 months	330	0.71 (0.19)	332	0.72 (0.19)	319	0.72 (0.19)	343	0.71 (0.19)
**Fear Avoidance Belief Questionnaire Physical Activity**								
Baseline	349	15·6 (5·6)	354	14·5 (5·4)	344	14·9 (5·7)	359	15·2 (5·3)
8 weeks	279	11·9 (6·4)	307	11·9 (6·0)	276	12·0 (6·3)	310	11·8 (6·1)
6 months	276	9·9 (6·5)	287	9·4 (6·4)	265	9·4 (6·6)	298	9·8 (6·3)
12 months	280	9·5 (6·6)	291	8·1 (6·8)	271	8·5 (6·9)	300	9·1 (6·5)
Over 12 months	324	10·4 (6·6)	332	9·8 (6·6)	316	10·0 (6·8)	340	10·2 (6·4)
**Pain Self-efficacy Questionnaire**								
Baseline	352	9·6 (2·4)	354	9·8 (2·3)	346	9·7 (2·3)	360	9·6 (2·3)
8 weeks	280	10·2 (2·4)	306	10·3 (2·2)	276	10·2 (2·3)	310	10·3 (2·3)
6 months	276	10·5 (2·1)	288	10·4 (2·2)	266	10·5 (2·1)	298	10·4 (2·2)
12 months	280	10·7 (2·0)	291	10·8 (2·2)	271	10·9 (1·8)	300	10·6 (2·3)
Over 12 months	325	10·5 (2·2)	332	10·5 (2·2)	317	10·5 (2·1)	340	10·4 (2·3)
**Insomnia Severity Index**								
Baseline	349	10·7 (6·4)	350	10·3 (6·1)	343	10·2 (6·3)	356	10·7 (6·3)
8 weeks	277	7·5 (6·2)	303	8·0 (6·1)	274	8·7 (6·3)	306	7·0 (6·0)
6 months	275	6·1 (5·8)	290	6·2 (6·1)	267	6·1 (5·9)	298	6·2 (6·0)
12 months	281	5·8 (6·1)	292	5·2 (5·7)	272	5·4 (5·9)	301	5·6 (6·0)

Over 12 months	323	6·5 (6·1)	329	6·5 (6·1)	314	6·7 (6·2)	338	6·3 (6·0)
**Return to Desired Activities**								
Baseline	352	8·1 (2·6)	354	7·7 (2·6)	346	7·8 (2·7)	360	7·9 (2·5)
8 weeks	278	6·2 (2·5)	303	6·2 (2·5)	274	6·5 (2·6)	307	5·9 (2·4)
6 months	276	5·4 (2·4)	290	5·0 (2·3)	268	5·1 (2·4)	298	5·2 (2·3)
12 months	281	4·8 (2·3)	292	4·6 (2·3)	273	4·6 (2·4)	300	4·8 (2·3)
Over 12 months	325	5·5 (2·5)	332	5·3 (2·5)	317	5·4 (2·6)	340	5·3 (2·4)
**Global Impression of Treatment**								
Baseline	..	..	..	..	..	..	..	..
8 weeks	277	7·6 (2·1)	304	7·8 (1·8)	274	7·3 (2·0)	307	8·1 (1·8)
6 months	276	8·2 (2·2)	291	8·7 (2·1)	269	8·5 (2·2)	298	8·5 (2·2)
12 months	283	8·6 (2·3)	293	9·1 (2·2)	274	8·9 (2·1)	302	8·8 (2·4)
Over 12 months	326	8·2 (2·2)	332	8·5 (2·1)	317	8·2 (2·2)	347	8·5 (2·1)

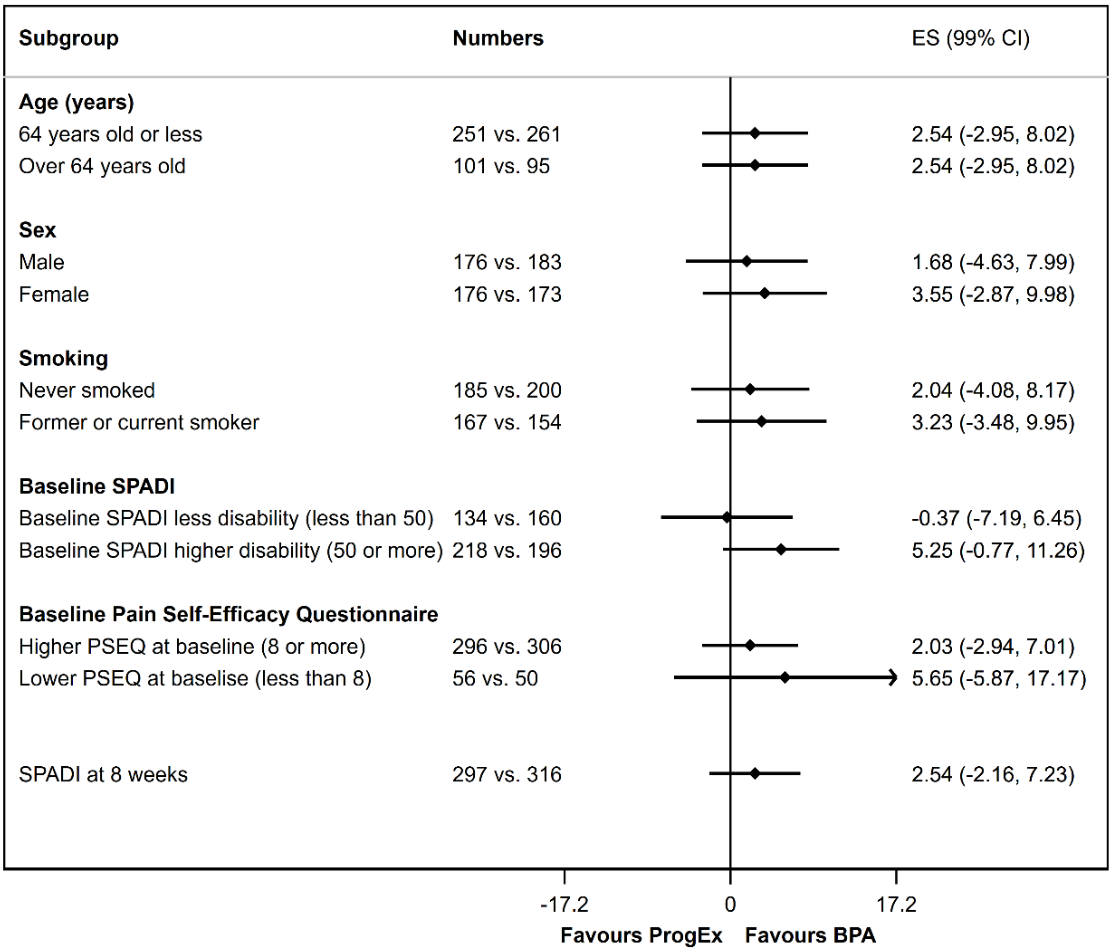

ES - Effect Size, CI- Confidence Interval

SUBGROUP ADJUSTED SPADI ANALYSIS FOR PROGRESSIVE EXERCISE VERSUS BEST PRACTICE ADVICE AT 8 WEEKS

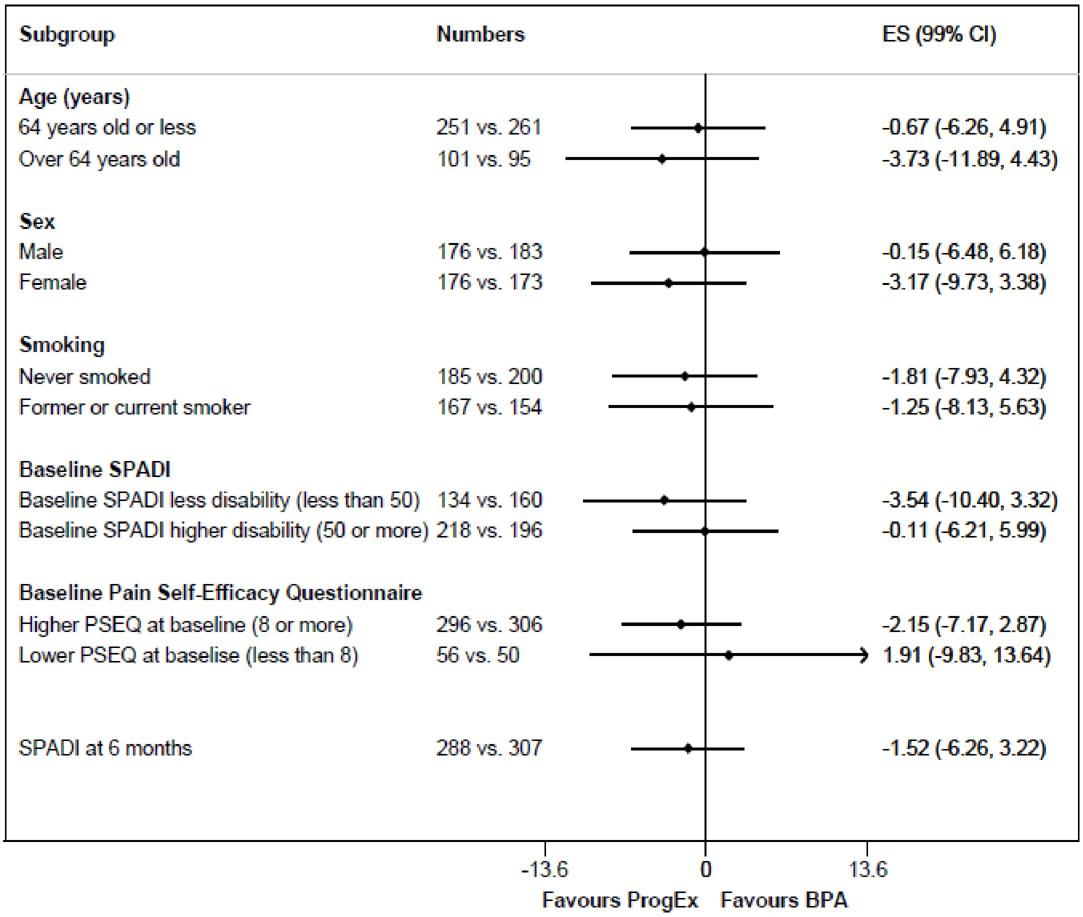

ES - Effect Size, CI- Confidence Interval

SUBGROUP ADJUSTED SPADI ANALYSIS FOR PROGRESSIVE EXERCISE VERSUS BEST PRACTICE ADVICE AT 6 MONTHS

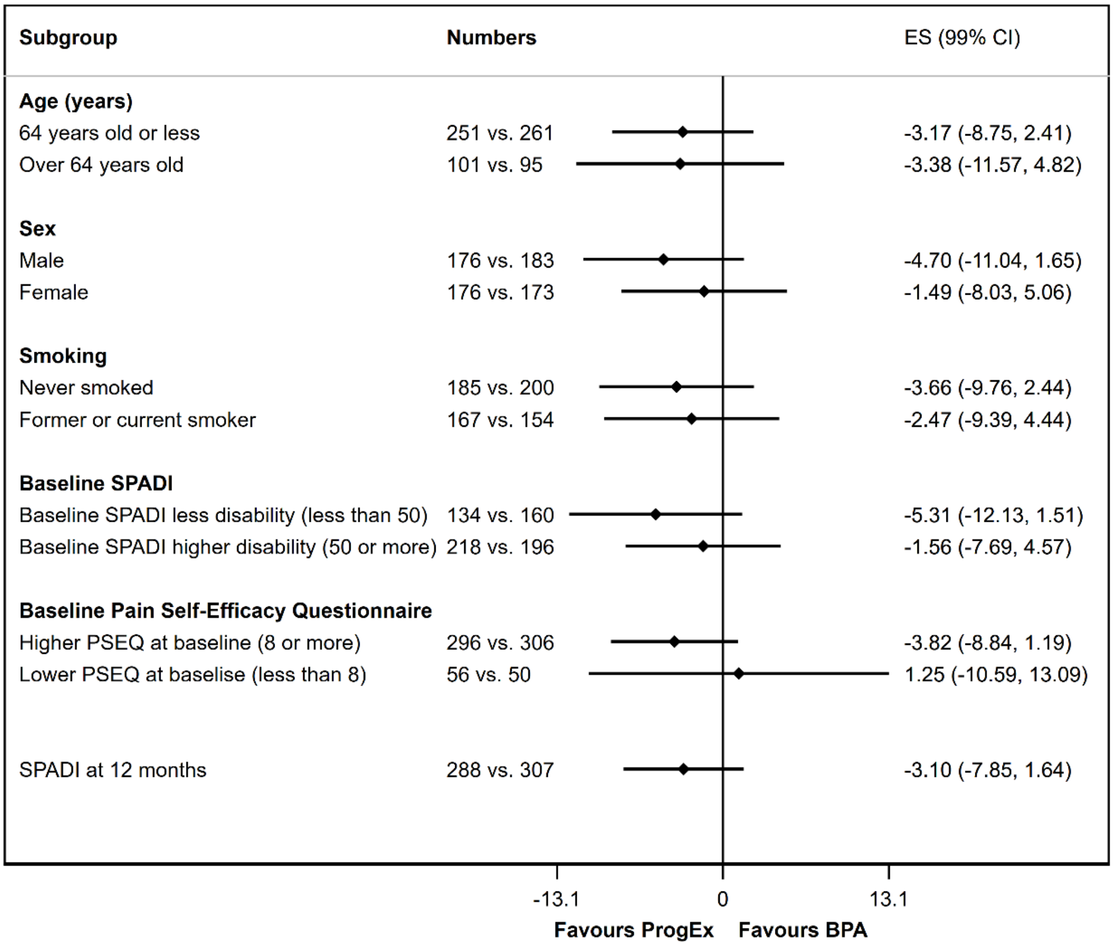

ES - Effect Size, CI- Confidence Interval

SUBGROUP ADJUSTED SPADI ANALYSIS FOR PROGRESSIVE EXERCISE VERSUS BEST PRACTICE ADVICE AT 12 MONTHS

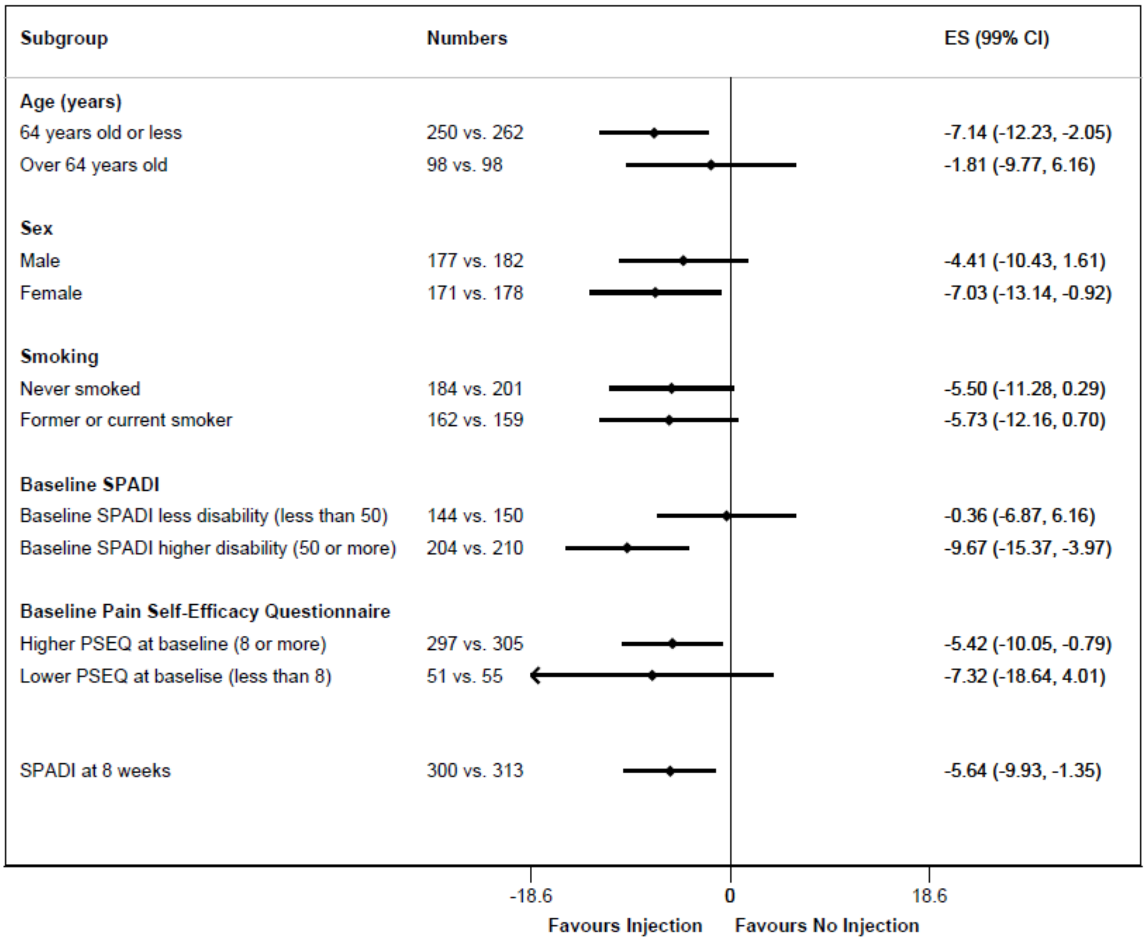

ES - Effect Size, CI- Confidence Interval

SUBGROUP ADJUSTED SPADI ANALYSIS FOR INJECTION VERSUS NO INJECTION AT 8 WEEKS

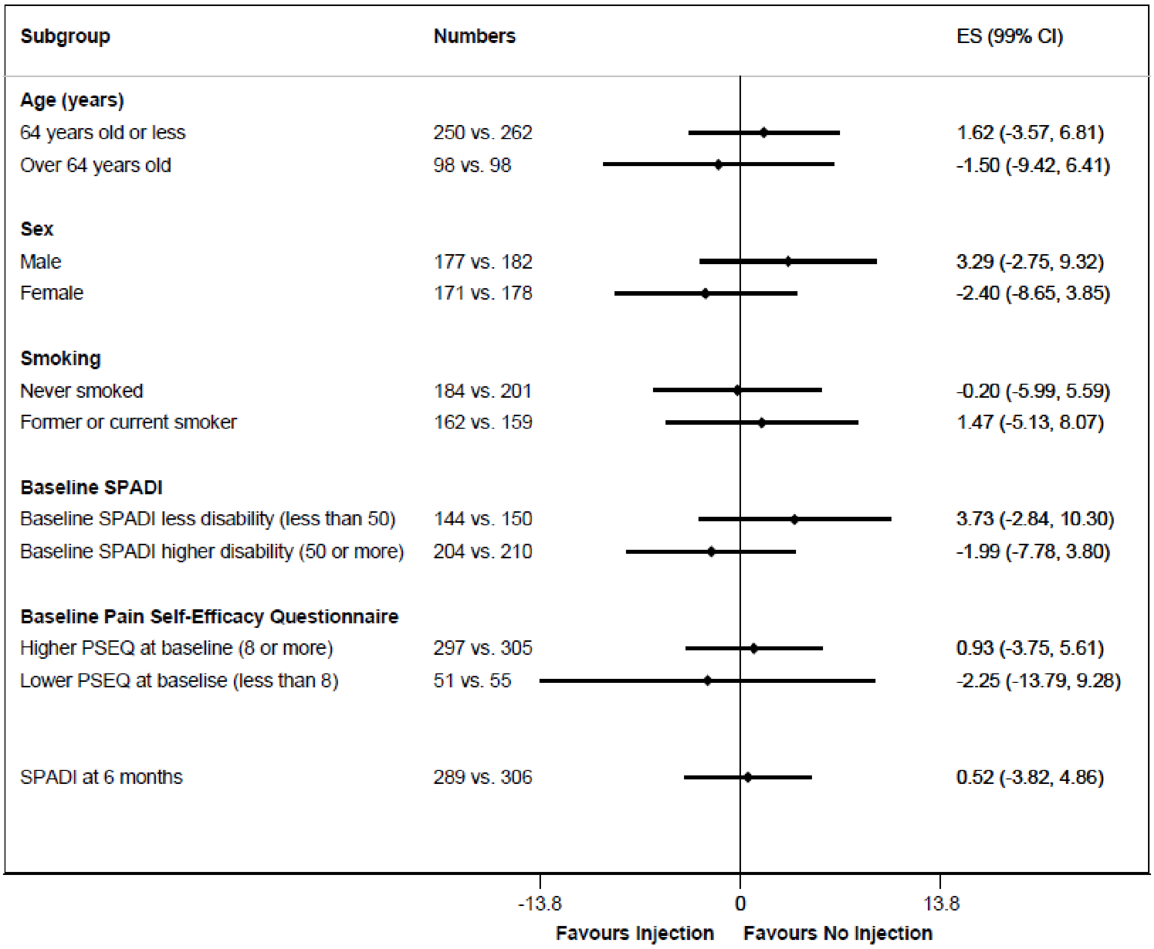

ES - Effect Size, CI- Confidence Interval

SUBGROUP ADJUSTED SPADI ANALYSIS FOR INJECTION VERSUS NO INJECTION AT 6 MONTHS

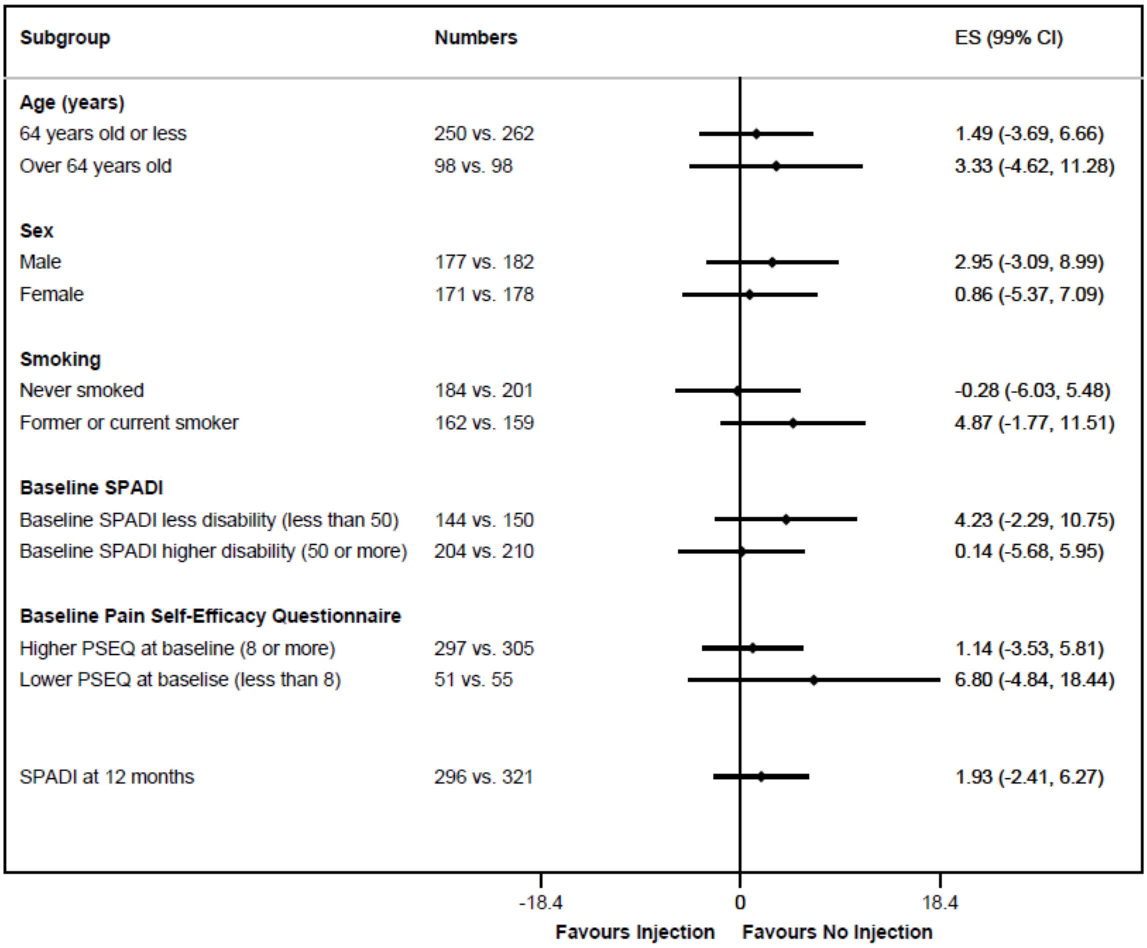

ES - Effect Size, CI- Confidence Interval

Subgroup adjusted SPADI analysis for injection versus no injection at 12 months

REGRESSION ANALYSIS WITH AN INTERACTION TERM, INCLUDING IMPUTATION OF MISSING VALUES AND ADJUSTMENT FOR BASELINE UTILITY, SEX AND AGE (BASE CASE ANALYSIS)

		Total costs mean (SE)	QALYs mean (SE)	NMB‡ mean (SE)
**Best Practice Advice (B)**		£195 (£54)	0·737 (0·013)	£14,538 (£290)
**Injection + Best Practice Advice (IB)**		£205 (£20)	0·757 (0·011)	£14,939 (£227)
**Progressive Exercise (P)**		£247 (£23)	0·756 (0·012)	£14,865 (£255)
**Injection + Progressive Exercise (IP)**		£307 (£30)	0·742 (0·012)	£14,524 (£263)
**Injection simple effect (IB-B)**		£10 (£44) (p=0·747)	0·021 (0·015) (p=0·184)	£402 (£322) (p=0·212)
**Progressive Exercise simple effect (P-B)**		£52 (£44) (p=0·247)	0·019 (0·016) (p=0·220)	£327 (£323) (p=0·309)
**Interaction (B-IB-P+IP)**	**Progressive** Exercise by injection	£50 (£57) (p=0·397)	-0·035 (0·022) (p=0·106)	-£743 (£455) (p=0·100)

Values represent the mean (standard error) for each group for males of age 55.46 years and a baseline utility of 0.653; since there was assumed to be no interaction between baseline variables and treatments, the simple effects for each treatment and the interaction between treatments are assumed to be the same for all participant subgroups, although the absolute costs and absolute QALYs may be higher or lower depending on participants' sex*, age and baseline utility.*

† Net monetary benefit calculated at a ceiling ratio of £20,000 per quality-adjusted life-year (QALY).

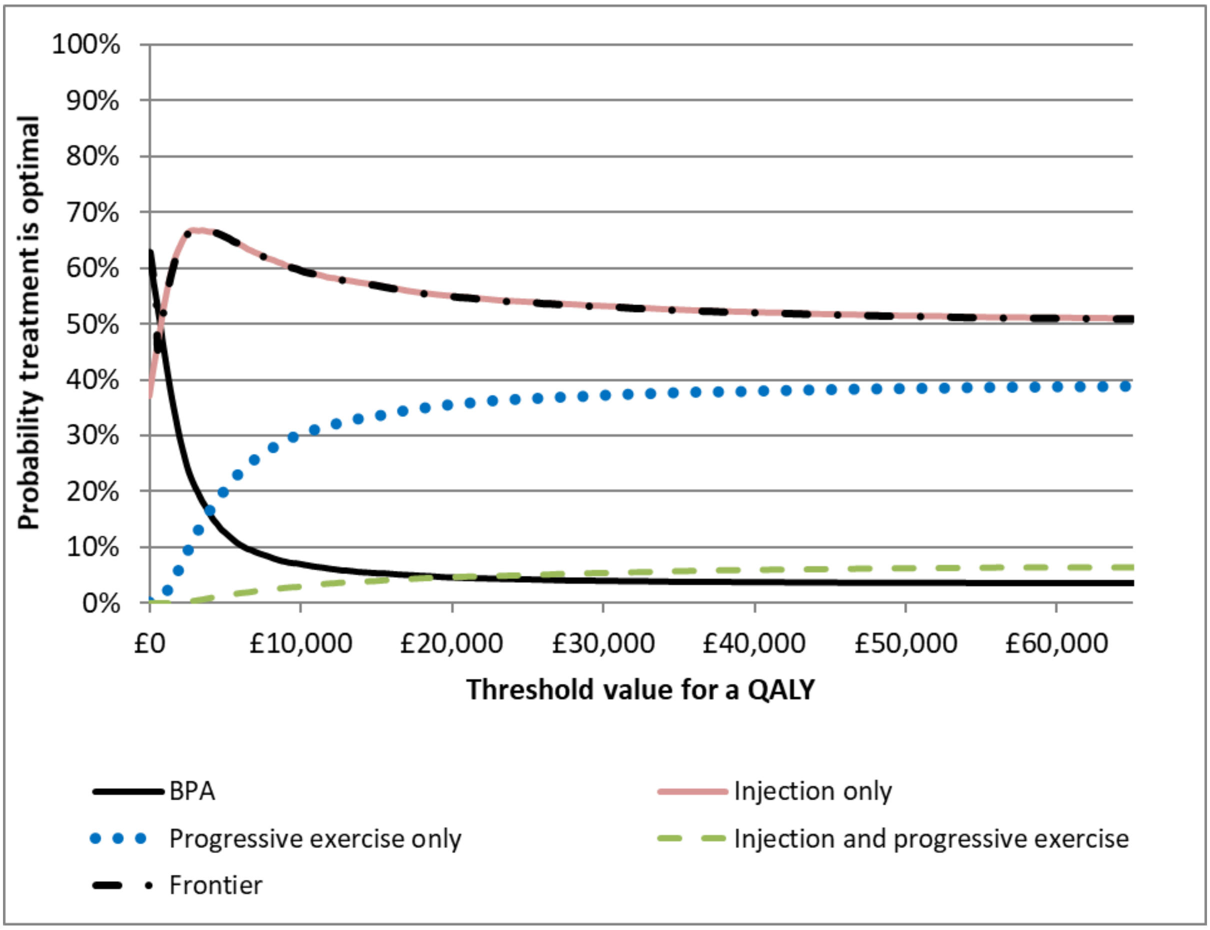

**BPA= Best Practice Advice; ** The Frontier indicated which treatment is economically preferred at different threshold values for cost-effectiveness*

COST-EFFECTIVENESS ACCEPTABILITY CURVE FOR THE COMPARISON BETWEEN TREATMENT GROUPS (BASE CASE ANALYSIS)

MEDLINE SEARCH STRATEGY

Database: Medline (Ovid MEDLINE® Epub Ahead of Print, In-Process & Other Non-Indexed Citations, Ovid MEDLINE® Daily and Ovid MEDLINE®) 1946 to present
Search Strategy:
----
1 Shoulder Pain/ (4378)
2 Shoulder Impingement Syndrome/ (1655)
3 Rotator Cuff/ and Tendinopathy/ (477)
4 Shoulder/ and (Bursitis/ or Tendinopathy/) (438)
5 ((shoulder* or rotator cuff or subacromial or sub-acromial) adj5 (bursitis or impinge* or tendinitis or tendonitis or tendinopathy or pain*)).ti,ab. (12483)
6 1 or 2 or 3 or 4 or 5 (14765)
7 (Cortisone/ or Glucocorticoids/) and Injections/ (1354)
8 ((subacromial or sub-acromial or corticosteroid* or cortisone or glucocorticoid*) adj5 inject*).ti,ab. (5093)
9 CSI.ti,ab. (3029)
10 7 or 8 or 9 (9227)
11 6 and 10 (480)
12 randomized controlled trial.pt. (477274)
13 controlled clinical trial.pt. (92948)
14 (randomized or randomised).ab. (522076)
15 placebo.ab. (195900)
16 drug therapy.fs. (2088506)
17 randomly.ab. (306731)
18 trial.ab. (455979)
19 groups.ab. (1887811)
20 12 or 13 or 14 or 15 or 16 or 17 or 18 or 19 (4404372)
21 exp animals/ not humans.sh. (4554611)
22 20 not 21 (3810278)
23 11 and 22 (320)
